# The critical role of FXR is associated with the regulation of autophagy and apoptosis in the progression of AKI to CKD

**DOI:** 10.1038/s41419-021-03620-z

**Published:** 2021-03-25

**Authors:** Dong-Hyun Kim, Jung Sun Park, Hoon-In Choi, Chang Seong Kim, Eun Hui Bae, Seong Kwon Ma, Soo Wan Kim

**Affiliations:** Department of Internal Medicine, Chonnam National University Medical School, Gwangju, Republic of Korea

**Keywords:** Transcriptional regulatory elements, Acute kidney injury

## Abstract

Autophagy is important for cells to break down and recycle cellular proteins, remove damaged organelles, and especially, for recovery from acute kidney injury (AKI). Despite research on the role and cellular mechanism of autophagy in AKI, the role of autophagy in the progression to chronic kidney disease (CKD) remains poorly understood. Here, using farnesoid X receptor (FXR) knockout (KO) mice, we determined whether FXR prevents the progression of AKI to CKD after renal ischemic-reperfusion (such as I/R) injury through the regulation of renal autophagy and apoptosis. FXR regulated genes that participate in renal autophagy under feeding and fasting conditions, such as hepatic autophagy, and the activation of FXR by agonists, such as GW4064 and INT-747, attenuated the increased autophagy and apoptosis of hypoxia-induced human renal proximal tubule epithelial (HK2) cells. The expression levels of autophagy-related and apoptosis-related proteins in FXR KO mice were increased compared with those in wild-type (WT) mice. We also showed that the increase in reactive oxidative species (ROS) in hypoxia-treated HK2 cells was attenuated by treatment with FXR agonist or by FXR overexpression, and that the level of ROS was elevated in FXR-deficient cells and mice. At 28 days after I/R injury, the autophagy levels were still elevated in FXR KO mice, and the expression levels of fibrosis-related proteins and ROS deposits were higher than those in WT mice. In conclusion, the regulation of renal autophagy and apoptosis by FXR may be a therapeutic target for the early stages of kidney damage, and the progression of AKI to CKD.

## Introduction

AKI, which is defined as sudden kidney failure followed by sudden kidney damage, is independently associated with an increased risk of death and the development of CKD and end stage renal disease (ESRD)^1,2^. Recent epidemiological and experimental studies have demonstrated that AKI contributes to the development and progression of CKD. In particular, the induction of autophagy in tubular epithelial cells has been documented in rodent models of AKI induced by renal I/R injury, nephrotoxic drugs, such as cisplatin, sepsis, and other AKI risk factors^3,4^. In addition, AKI induces cell death pathway activation through apoptotic and necrotic mechanisms in tubular epithelial cells^5^. Despite these studies, the principles of the regulatory mechanisms of autophagy and apoptosis in the progression from AKI to CKD are poorly understood.

Macroautophagy (hereafter termed autophagy) is a normal cellular homeostatic process that removes dysfunctional components and damaged organelles, and recycles cellular proteins through the lysosomal degradation pathway^6,7^. Autophagy, which is associated with several diseases and must be tightly controlled to maintain cellular homeostasis, is known to occur based on the response to either intracellular or extracellular factors, such as extremely stressful starvation conditions, hypoxia, endoplasmic reticulum stress or oxidative stress, organelle damage, and pathogen infections^8–13^. It has been reported that a deficiency or excess of autophagy-related proteins contributes to several diseases. Selective deletion of Atg5 or Atg7 in the proximal tubules of mice resulted in progressive kidney damage, and increased tubular cell apoptosis and tubulointerstitial fibrosis compared with WT mice^14–16^. In addition, after I/R injury, tightly controlled autophagy is important to maintain the viability of renal tubules after initial damage, and because this process contributes to normal kidney repair and regeneration^4^.

Farnesoid X receptor (FXR, NR1H4) is a member of the nuclear receptor superfamily of ligand-activated transcription factors, and plays a key role in maintaining cholesterol and bile acid levels^17,18^. FXR is highly expressed in the liver and intestine and especially in the kidneys, where it is mainly found in proximal tubular epithelial cells. In renal disease, FXR has been shown to have anti‐inflammatory, antifibrotic, antilipogenic, and antioxidant functions. FXR activation has been shown to interfere with renal overexpression of SREBP-1 and lipogenic enzymes, inhibit the nuclear factor kappaB (NF-κB) pathway, and protect against renal fibrosis by suppressing SMAD3 expression and regulating the FXR-Src-YAP pathway^19–22^. In addition, FXR epigenetically controls the transcription of hepatic autophagy genes. However, the study of renal autophagy regulation by FXR is not well known, and a study on the effect of autophagy regulation by FXR on the progression from AKI to CKD is needed.

In this study, we investigated the regulation of renal autophagy and apoptosis by FXR using hypoxia-treated HK2 cells and an I/R injury model in FXR-deficient mice. We demonstrated that renal autophagy is regulated by FXR activation, such as in hepatic autophagy, and that FXR deficiency increased the levels of autophagy and apoptosis through the induction of ROS. Furthermore, we revealed the critical role of FXR in CKD progression post-AKI, which indicates that FXR may be a pharmacological target in the early stages of kidney damage and that it can protect against the progression of AKI to CKD.

## Materials and methods

### Materials

Antibodies for FXR (sc-13063), ATG3 (sc-393660), and GAPDH (sc-32233) were purchased from Santa Cruz Biotechnology and for ATG7 (8558), BECN1 (3738), LC3 (4108), Actin (4970), ATG16L1 (8089), SQSTM1/p62 (5114), Bax (2772), Bcl2 (3498), Cleaved caspase 3 (9611), and 3-nitro tyrosine (9691) from Cell Signaling. Antibodies for M2 (F3165) was purchased from Sigma, Inc. F4/80 (MCA497GA) was purchased from Bio-Rad. GW4064 and WAY-362450 was obtained from Tocris bioscience and AdooQ Bioscience, respectively. INT-747 and Picro Sirius Red Stain Kit (ab150681) were obtained from Abcam. N-TARGETplus human siRNAs for FXR (L-003414) and ATG7 (L-020112) were purchased from GE Healthcare Dharmacon, Inc. ptfLC3 was a gift from Tamotsu Yoshimori (Addgene plasmid # 21074; http://n2t.net/addgene:21074; RRID:Addgene_21074)^23^.

### Animal experiments

Eight-week-old male C57BL6 and FXR KO mice (JAX stock #004144) were purchased by Samtako (Korea) and Jackson laboratory (Bar Harbor, ME, USA), respectively. Mice were anesthetized with ketamine (50 mg/kg; Yuhan, Seoul, Korea) and xylazine (10 mg/kg; Bayer Korea) intraperitoneally and placed on a temperature-regulated table (37 °C) to maintain body temperature. Renal ischemia was induced by clamping both renal pedicles with micro clamp (ROBOZ, Gaithersburg, USA) for 30 min. I/R group (*n* = 6–8) was sacrificed after 7 or 28 days of reperfusion. Control group (*n* = 6) underwent the same procedure, except that the clamp was not applied. All animal experiments were approved by the Animal Care Regulations (ACR) Committee of Chonnam National University Medical School, and our protocols conformed to the institution guidelines for experimental animal care and use.

### Cell culture

Human renal proximal tubular epithelial cells (HK2, ATCC, Manassas, VA, USA) was cultured in Dulbecco’s modified Eagle’s Medium-F-12 (DMEM-F12) (WelGene, Daegu, Korea) supplemented with 10% fetal bovine serum, 100 U/mL penicillin, and 100 μg/mL streptomycin at 37 °C under a humidified 5% CO_2_ atmosphere. For hypoxic culture, HK2 cells were incubated in a humidified modular incubator chamber (Modular Incubator Chamber, Billups-Rothenberg inc) at 37 °C in an atmosphere of 1% O_2_, 5% CO_2_ and 94% N_2_ for 0, 3, 6, 9, or 16 h, followed by 12 h of reoxygenation (21% O_2_, 5% CO_2_, 37 °C). Control cells (normoxia cells) were incubated for equivalent periods under normoxic conditions (21% O_2_, 5% CO_2_, 37 °C). For immunofluorescence experiment, HK2 cells were transfected with expression plasmids for mRFP-GFP-LC3 (ptfLC3) and 48 h later were incubated in hypoxia chamber for 6 h. Counterstained with DAPI and were imaged with LSM 800 confocal laser scanning microscopy.

### siRNA knockdown

RNA interference of FXR and ATG7 were performed using an FXR-specific, and ATG7-specific siRNA from Dhamacon ON-TARGETplus. Briefly, cells were transfected with indicated concentration of siRNA (30 nM) using DhamaFECT 2 transfection reagent according to the manufacturer’s protocol. Cells transfected with control siRNA (ON-TARGETplus Non-Targeting Pool) were used as controls for direct comparison.

### Western blot analysis

Cell extracts were prepared by brief sonication of cell pellets in RIPA buffer containing 50 mM Tris-HCl, pH 7.6, 150 mM NaCl, 5 mM EDTA, 1% NP40, 0.1% SDS, and protease inhibitors. Kidney tissue was homogenized in RIPA buffer. Cell extracts and tissue homogenates were centrifuged at 1500×*g* for 20 min at 4 °C to remove cell debris. Immunoblotting was carried out as previously described^24^. Target proteins were detected using indicated antibodies by immunoblotting. Quantitative analysis of band intensity was performed using imageJ software (National Institutes of Health, Bethesda, MD, USA).

### Kidney histologic analysis

Kidneys were fixed with PBS containing 4% paraformaldehyde, and then embedded in paraffin sections were used in immunohistochemical (IHC) staining and H&E. IHC staining was performed using indicated antibodies and horseradish peroxidase-conjugated anti-mouse or anti-rabbit IgG secondary antibodies (Dako). The stained sections were imaged with Nikon Eclipse Ni-U microscope (Tokyo, Japan). The quantitative analysis of stained section was performed using imageJ software (National Institutes of Health, Bethesda, MD, USA).

### Quantification of mRNA

To quantify mRNA levels, total RNA was extracted from mouse kidney and liver, or HK2 cells using TRIzol reagent (Invitrogen). cDNA was then reverse transcribed from 1 μg samples of total RNA using AMV Reverse Transcription System (Promega Corp., Madison, WI, USA). Real-time PCR was performed using SYBR Green PCR master mix (Thermo Fisher Scientific, Austin, USA) and StepOnePlus Real-Time PCR System (Thermo Fisher Scientific, Austin, USA). The list of qRT–PCR primer sequences is shown in Supplementary Table 1.

### TUNEL staining

Apoptosis was determined using the ApopTag Plus Peroxidase In Situ Apoptosis Detection Kit (Chemicon International; Temecula, CA, USA) and DeadEnd™ Fluorometric TUNEL System (Promega Corporation) for in vivo and in cells according to the manufacturer’s protocol, respectively. The sections were counterstained with hematoxylin and examined by light microscopy.

### Cell viability assay

Cell viability was determined using the EZ-CyTox (tetrazolium salt, WST-1) cell viability assay kit (Daeil Lab Service, Seoul, Korea), as previously described^25^. HK2 cells were plated into 96-well tissue culture plates and allowed to grow for 48 h followed by treatment with vehicle or FXR agonists as indicated, and then cells were incubated in hypoxia-chamber for indicated time. Absorbance at 570 nm was detected using a 96-well microplate reader (BioTek Instruments, Winooski, VT, USA). Cell viability is expressed as the fraction of the surviving cells relative to the vehicle-treated cells.

### Measurement of ROS generation

Level of intracellular ROS was assessed using 5,6-chloromethyl-2′,7′-dichlorodihydrofluorescein diacetate (CM-H_2_DCFDA; Invitrogen, Carlsbad, CA, USA). After cells were treated with FXR agonists or transfected with siRNA and flag-FXR plasmid, and then cells were incubated in hypoxia chamber for 6 h. Normoxic and hypoxia induced cells were incubated with 10 μM CM-H_2_DCFH-DA and were immediately visualized using EVOS FL Auto Imaging System (Thermo fisher scientific) or the ROS levels were measured using Promega GloMax plate reader (Promega).

### Statistical analysis

GraphPad Prism 8 (GraphPad software version 8.01) was used for data analysis. Statistical significance was determined by one-way or two-way ANOVA with the Bonferroni post-test for single or multiple comparisons as appropriate. *P*-values < 0.05 were considered as statistically significant. All experiments were performed at least three times.

## Results

### FXR is downregulated in the I/R model and in hypoxia-treated HK2 cells

**Fig. 1:**
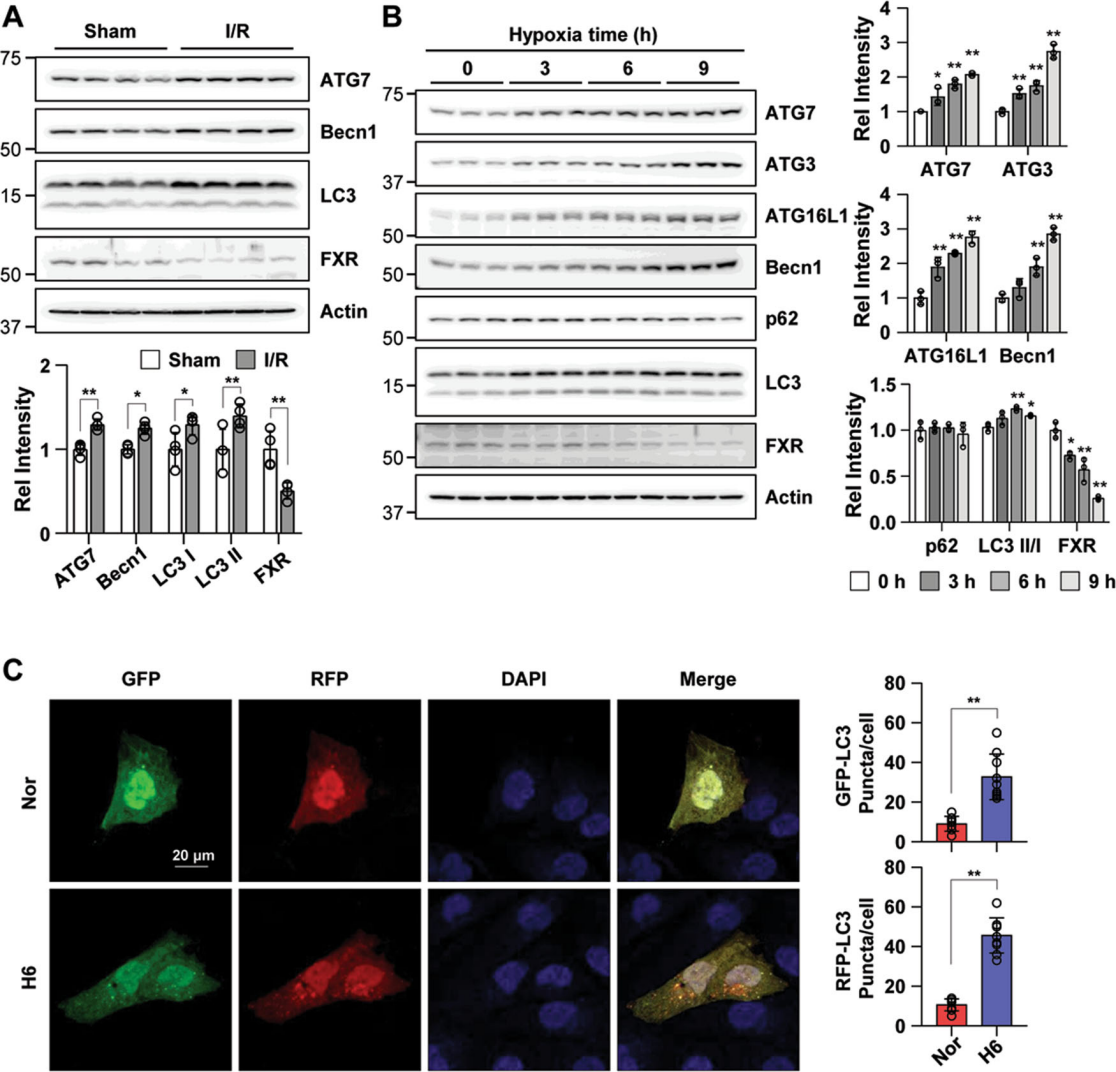
I/R injury and hypoxia increase renal autophagy and decrease FXR expression in mice and HK2 cells. **A** At 48 h after I/R, the mice were euthanized, and the kidneys were collected (*n* = 4). Protein levels of ATG7, Becn1, LC3, and FXR were detected by immunoblotting. The relative protein levels are shown. The values for the Sham group were set to 1. **B** HK2 cells were exposed to hypoxia for the indicated times, and the levels of autophagy-related proteins and FXR were detected by immunoblotting. The relative levels are shown (*n* = 3). The level of each protein, including actin, in the 0 h hypoxia sample was set to 1. **C** HK2 cells were transfected with the ptf-LC3 expression plasmid, after which the cells were subjected to hypoxic conditions for 6 h. Fluorescence was imaged by confocal microscopy. The average number of LC3 II puncta per cell is shown on the right (*n* = 8–9). Scale bar, 20 μm. All values are presented as the mean ± SD. Statistical significance was measured using one-way ANOVA with the Bonferroni post-test. *P < 0.05, **P < 0.005.

Autophagy is upregulated under stress conditions, including nutrient deprivation, energy-limiting cellular stress, hypoxia, and oxidant injury, and is part of the pathogenesis of acute kidney injury^3^. To investigate the involvement of FXR expression in I/R-induced kidney injury, we assessed the FXR expression pattern in an I/R injury mouse model. The expression levels of the autophagy-related proteins ATG7 and Becn1 and the LC3 II/I ratio were increased in the I/R injury group compared with the sham group. The expression level of FXR in the I/R injury group was markedly decreased compared with that in the sham group (Fig. 1A). To mimic I/R injury in HK2 cells, HK2 cells were incubated in a hypoxia chamber for the indicated times. The expression levels of the autophagy-related proteins ATG7, ATG3, ATG16L1, and Becn1 and the LC3 II/I ratio, were significantly increased in a time-dependent manner, while the expression level of FXR was decreased (Fig. 1B). The green and red fluorescent protein (mRFP-GFP)-LC3 puncta in HK2 cells exposed to hypoxia were significantly increased compared with those in normoxic HK2 cells (Fig. 1C). These results suggest that FXR was downregulated and the protein expression of autophagy-related proteins was increased in the I/R-induced AKI mouse model and hypoxia-treated HK2 cells.

### FXR inhibits renal autophagy in vivo

**Fig. 2:**
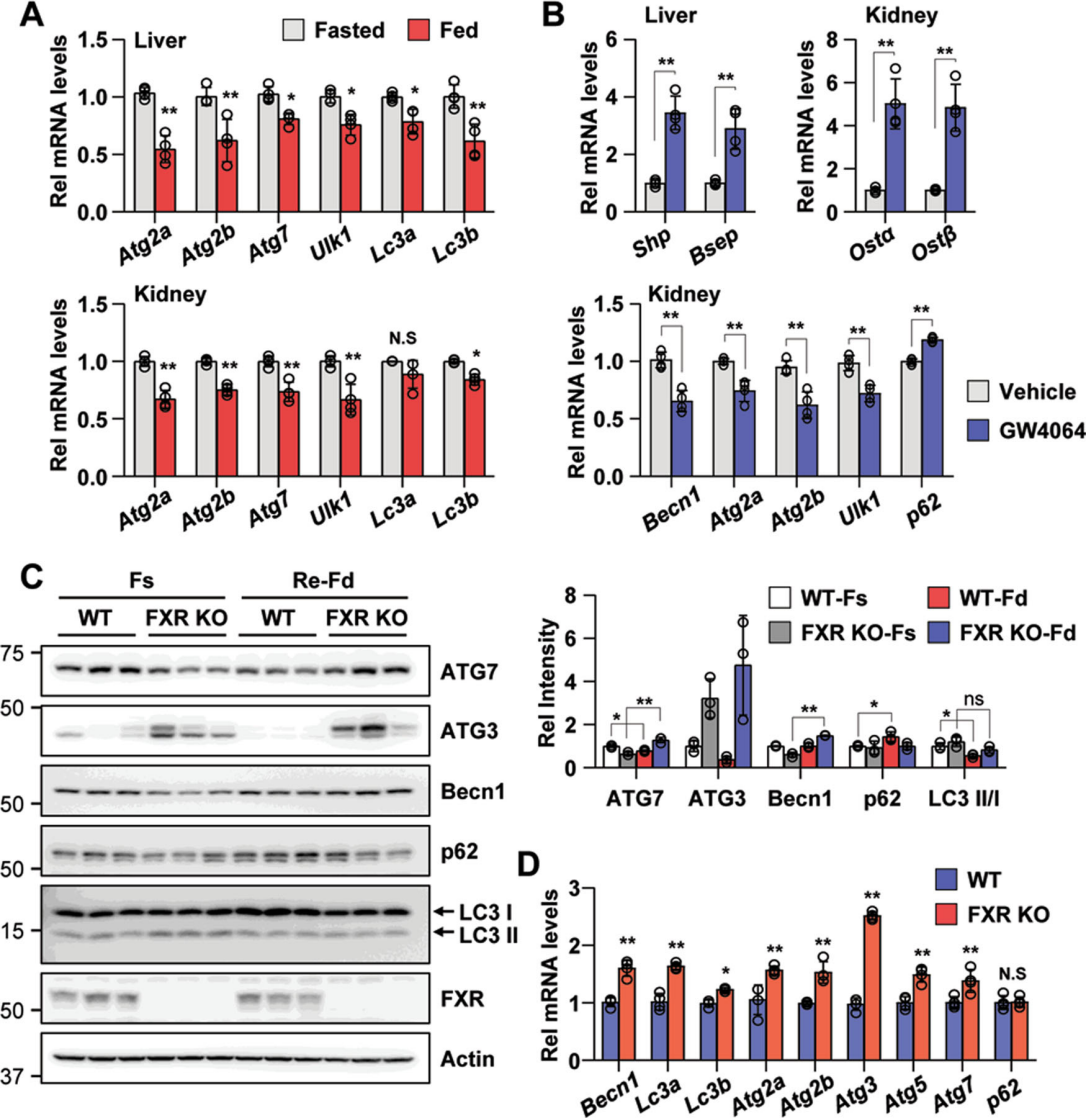
FXR inhibits renal autophagy by feeding and GW4064. **A**, **B** Wild-type mice were fasted for 12 h at which point were then fed a normal chow diet (**A**) and treated with vehicle or GW4064 for 6 h. The liver and kidneys were collected, and the mRNA levels of the indicated genes were measured by qRT-PCR. The values for fasted or vehicle treatment were set to 1 (*n* = 4). **C** Wild-type and FXR knockout mice were fasted and refed, and the kidneys were collected. The levels of autophagy-related proteins and FXR were detected by immunoblotting. The relative levels are shown (*n* = 3). The level of each protein, including actin, in the wild-type fasting sample was set to 1. **D** Wild-type and FXR knockout mice were fasted for 6 h, and the kidneys were collected. The mRNA levels of the indicated genes were measured by qRT-PCR. The values for wild-type mice were set to 1 (*n* = 4). All values are presented as the mean ± SD. Statistical significance was measured using one-way or two-way ANOVA with the Bonferroni post-test. *P < 0.05, **P < 0.005, ns statistically not significant, WT-Fs wild-type fasting, WT-Fd wild-type feeding, FXR KO-Fs FXR knockout fasting, FXR KO-Fd FXR knockout feeding.

Autophagy is known to be acutely regulated by nutrient-sensing cytoplasmic kinases^26^, but recent studies have demonstrated that it is regulated by nuclear events mediated by nutrient-sensing transcriptional factors, such as FXR, CREB, PPARα, and TFEB, under normal physiological conditions^12,27^. Since FXR regulates hepatic autophagy under physiological conditions, we tested whether FXR also regulates renal autophagy. To examine whether renal autophagy is regulated during feeding/fasting cycles under normal physiological conditions such as hepatic autophagy regulation, we tested the mRNA levels of autophagy-related genes in the liver and kidneys of fasting and feeding wild-type (WT) mice. The mRNA expression levels of the autophagy-related genes *Atg2a*, *Atg2b*, *Atg7*, *Ulk1*, *Lc3a*, and *Lc3b* in the liver after feeding were significantly decreased, as shown in previous studies. The mRNA levels of these genes were also significantly decreased in the kidney after feeding (Fig. 2A). To test the effects of pharmacological activation of FXR, we treated fasted mice with GW4064, a specific FXR agonist. Treatment with GW4064 in fasted mice increased the mRNA levels of a known FXR target genes, *Shp* and *Bsep* (liver), *Ostα* and *Ostβ* (kidney). Treatment with GW4064 decreased the mRNA level of the renal autophagy-related genes, *Becn1*, *Atg2a*, *Atg2b*, and *Ulk1*, and increased the *p62* mRNA level (Fig. 2B).

Next, to determine the physiological relevance of FXR-dependent regulation of renal autophagy, we tested whether FXR is required for feeding-mediated renal autophagy regulation through comparative experiments in WT and FXR knockout (FXR KO) mice. The expression levels of the autophagy-related proteins ATG7 and ATG3 and the LC3 II/I ratio were decreased in WT mouse kidneys after feeding, while the expression levels of these proteins and the LC3 II/I ratio in FXR KO mouse kidneys were increased or did not change. The p62 expression level was increased in WT mouse kidneys after feeding but did not change in FXR KO mouse kidneys (Fig. 2C). The mRNA levels of autophagy-related genes in fasted FXR KO mouse kidneys were also significantly increased compared with those in WT mouse kidneys (Fig. 2D). Consistent with previously reported hepatic autophagy regulation studies, these data suggest that renal autophagy is also regulated under physiological conditions, and renal autophagy is FXR-dependently regulated. Thus, it suggests that FXR probably plays an important role in the negative regulation of renal autophagy.

### Pharmacological FXR activation inhibits renal autophagy in hypoxia-treated HK2 cells

**Fig. 3:**
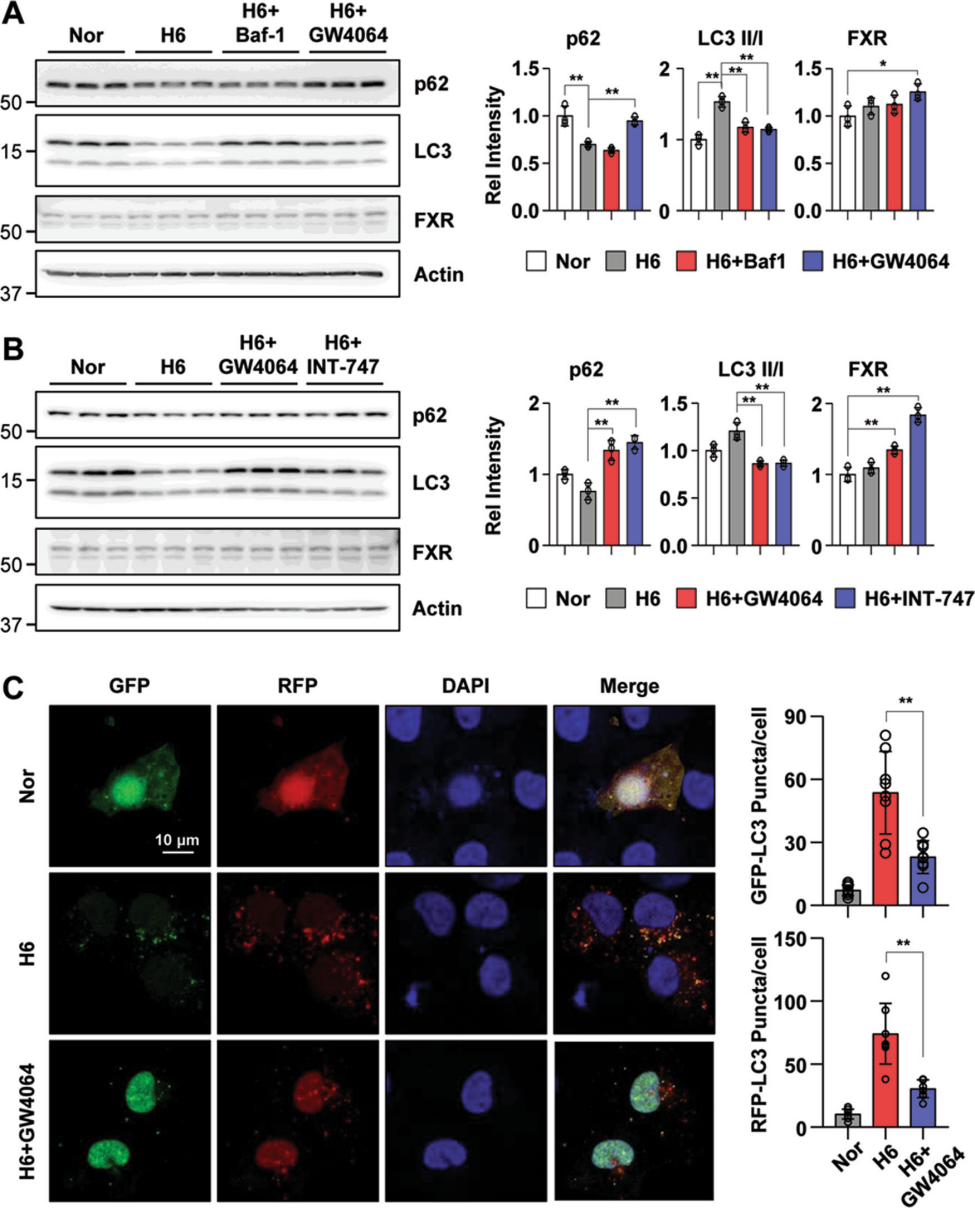
Autophagy is inhibited by FXR activation in hypoxia-treated HK2 cells. **A**, **B** After treatment with Baf-1 (100 nM), GW4064 (500 nM), or INT-747 (500 nM) for 1 h, HK2 cells were exposed to hypoxia for another 6 h. The protein levels of p62, LC3, and FXR were detected by immunoblotting, and the relative levels of p62/Actin, FXR/Actin, and the LC3 II/I ratio are shown in the right top and right bottom panels, respectively (*n* = 3). **C** HK2 cells were transfected with the ptf-LC3 expression plasmid. After treatment with GW4064 (500 nM) for 1 h, HK2 cells were exposed to with hypoxia for another 6 h. Fluorescence was imaged by confocal microscopy. The average number of LC3 puncta per cell is shown on the right (*n* = 8). All values are presented as the mean ± SD. Statistical significance was measured using one-way ANOVA with the Bonferroni post-test. *P < 0.05, **P < 0.005.

To test the effect of the FXR agonist GW4064 on the regulation of renal autophagy, we treated hypoxia-exposed HK2 cells with the autophagy inhibitors Baf-1 and GW4064. We examined the expression levels of LC3 and p62, which are markers of autophagy. p62/SQSTM1 is an autophagosome adapter protein that is degraded by autophagy, whereas p62 accumulates when autophagy is inhibited. Hypoxia-treated HK2 cells led to a decrease in the p62 expression level and an increase in the LC3 II/I ratio, while GW4064 treatment led to an increase in the p62 levels and a decrease in the LC3 II/I ratio (Fig. 3A). Additionally, we examined the inhibition of renal autophagy by pharmacological FXR activation using GW4064 and INT-747. Vehicle treatment in hypoxia-treated HK2 cells decreased the p62 expression level and increased the LC3 II/I ratio, whereas these changes were markedly reversed by GW4064 and INT-747 treatment (Fig. 3B). The FXR expression levels were increased by FXR agonists, GW406 and INT-747. To test the effect of antagonist and agonist of FXR on renal autophagy regulation, we examined the mRNA level of autophagy-related genes in hypoxia-induced HK2 cells after treatment with FXR antagonist (Z-guggulsterone) and agonists (GW4064 and INT-747). The mRNA levels of autophagy-related genes increased by hypoxia were significantly decreased by FXR agonists treatment, but not with antagonist treatment (Supplementary Fig. 1). Next, to determine autophagic flux in hypoxia-treated HK2 cells dependent on GW4064 treatment, we transfected HK2 cells with the mRFP-GFP-LC3 construct. Compared with the normoxic HK2 cells, hypoxia treatment induced a marked increase in both green and red puncta. Moreover, the increase in green and red puncta induced by hypoxia was significantly attenuated by GW4064 treatment (Fig. 3C). Collectively, these data suggest that hypoxia-induced autophagic flux could be inhibited by pharmacological FXR activation.

### FXR has a critical role in renal autophagy

**Fig. 4:**
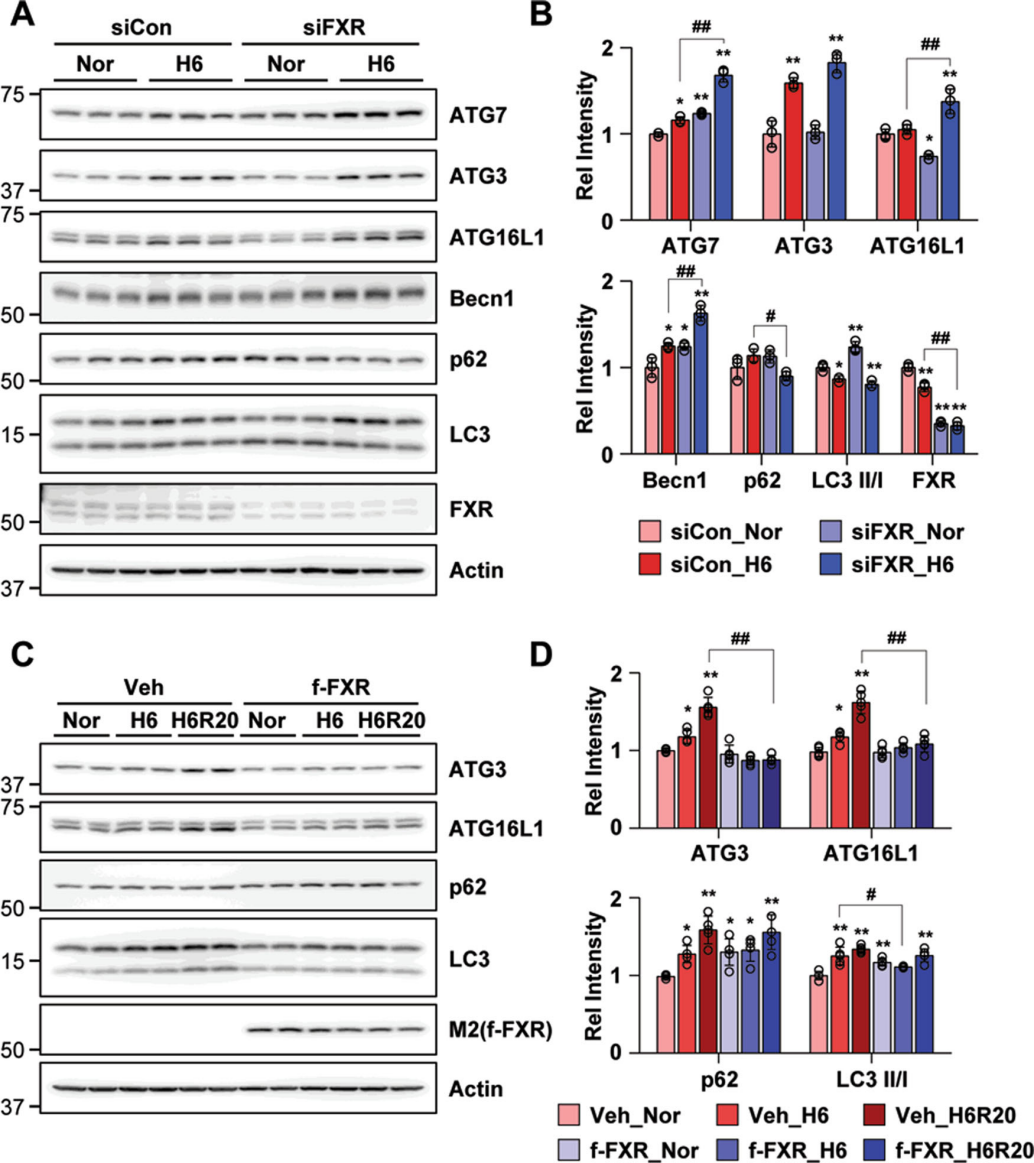
FXR is important for the regulation of renal autophagy. **A**–**D** HK2 cells were transfected with the siFXR (**A**, **B**) or FXR (**C**, **D**) expression plasmids as indicated, and 48 h later, the cells were exposed to hypoxia for 6 h. The protein levels of the indicated genes were detected by immunoblotting (**B**, **D**), and the relative protein levels are shown (*n* = 3–4). The values for the siControl normoxia (**B**) and vehicle normoxia (**D**) were set to 1. All values are presented as the mean ± SD. Statistical significance was measured using two-way ANOVA with the Bonferroni post-test. *P < 0.05, **P < 0.005, ^#^*P* < 0.05, ^##^*P* < 0.005.

Since pharmacologically activated FXR inhibited renal autophagy in hypoxia-treated HK2 cells, we examined the direct effects of FXR downregulation on the regulation of renal autophagy. FXR was downregulated in HK2 cells by siRNA. In siFXR normoxic HK2 cells, expression levels of ATG7 and Becn1 as well as the LC3 II/I ratio were markedly increased compared with the siControl normoxic HK2 cells (Fig. 4A, B). The expression levels of ATG7, ATG3, ATG16L1, and Becn1 after treatment of siControl cells with hypoxia were increased to a greater extent in siFXR hypoxia-treated HK2 cells, while the expression level of p62 was significantly decreased in siFXR hypoxia-treated HK2 cells. Furthermore, to comprehensively explore the effect on autophagy of FXR, we performed the experiment using autophagy inhibitors (Baf-1 and CQ) in FXR downregulated HK2 cells by siRNA. The downregulation of FXR in HK2 cells by siRNA markedly increased the expression level of ATG3, ATG7, and LC3 II, while the level of p62 was decreased. Compared to the siCon group, the increased LC3 expression levels in the siFXR group were further increased when treated with autophagy inhibitors (Supplementary Fig. 2). Additionally, the increased expression levels of ATG3 and ATG16L1 observed in hypoxia-treated and hypoxia-reoxygenation-treated HK2 cells were not observed in the FXR overexpression groups. FXR overexpression in HK2 cells led to increased p62 in normoxic cells compared with vehicle-expressing cells, while the increased LC3 II/I ratio in vehicle-expressing cells was markedly decreased by FXR overexpression in hypoxia-treated HK2 cells (Fig. 4C, D). These results suggest that the autophagy flux is increased in FXR-deficient HK2 cells, and that FXR directly affects the regulation of autophagy-related proteins induced by hypoxia.

### FXR deficiency increases renal apoptosis in vitro and in vivo

**Fig. 5:**
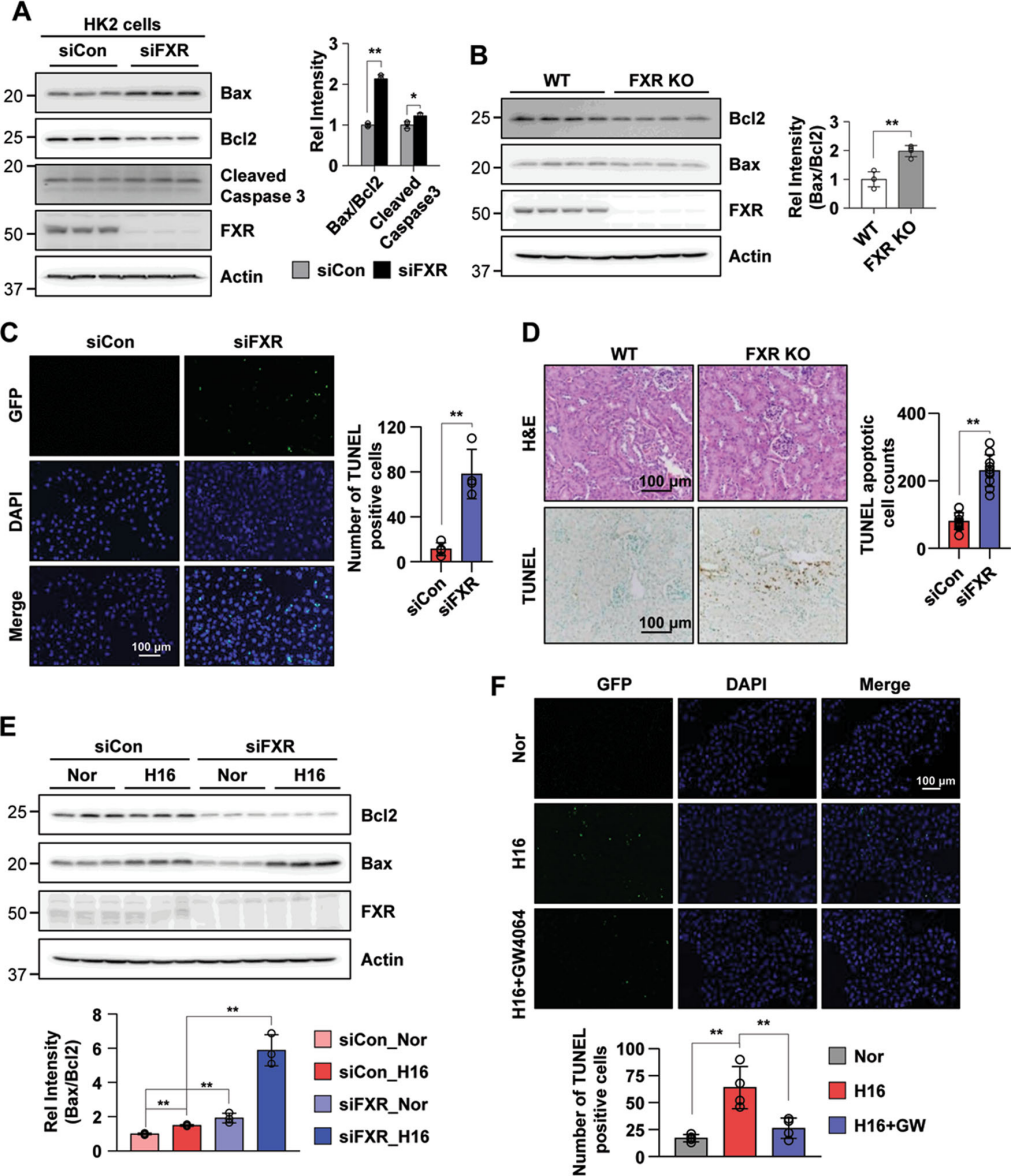
FXR deficiency increases renal apoptosis. **A**–**D** Comparison of the protein expression levels of molecules related to apoptosis, as determined by immunoblotting (**A**, **B**) and TUNEL staining (**C**, **D**) in HK2 cells transfected with siRNA against FXR (**A**, **C**) and in the kidneys of WT and FXR KO mice (**B**, **D**). The relative protein levels are shown (right panel of **A**, **B**) (*n* = 3–4). The values for the siControl and WT were set to 1. Quantitative analysis of positive TUNEL staining (right panel of **C**, **D**) (*n* = 4–11). **E** HK2 cells were transfected with siRNA against FXR as indicated, and 48 h later, the cells were exposed to hypoxia for 16 h. The protein levels of the indicated genes were detected by immunoblotting, and the relative intensity of Bax/Bcl2 is shown. The value for the siControl normoxia was set to 1 (*n* = 3). **F** Representative image of TUNEL staining. After treatment with GW4064 (500 nM) for 1 h, HK2 cells were exposed to hypoxia for another 16 h. Quantitative analysis of positive TUNEL staining is shown (*n* = 4). All values are presented as the mean ± SD. Statistical significance was measured using one-way or two-way ANOVA with the Bonferroni post-test. *P < 0.05, **P < 0.005. Nor normoxia, H16 hypoxia 16 h.

Since apoptosis is known to be induced in I/R injury and in hypoxia-treated cells^28,29^, we tested apoptotic events in an I/R injury mouse model and in hypoxia-treated HK2 cells. We confirmed a time-dependent increase in the Bax/Bcl-2 protein ratio in the I/R injury mice and in HK2 cells exposed to hypoxia (Supplementary Fig. 3). Next, we tested whether FXR deficiency induces apoptotic events in vitro and in vivo. The downregulation of FXR in HK2 cells by siRNA increased the Bax/Bcl-2 protein ratio and the cleaved caspase-3 level compared with siControl HK2 cells (Fig. 5A), and the Bax/Bcl-2 protein ratio in the FXR KO mice was increased compared with the WT mice (Fig. 5B). In the viability assay, pretreatment with the FXR agonists GW4064 or WAY-362450 increased cell viability in a dose-dependent manner in hypoxia-treated HK2 cells (Supplementary Fig. 4). We then performed a terminal deoxynucleotidyl transferase dUTP nick end labeling (TUNEL) assay to determine the degree of renal tubular apoptosis in vitro and in vivo. The numbers of tubular epithelial cells with TUNEL-positive nuclei in FXR-downregulated HK2 cells and in FXR KO mice were increased (Fig. 5C, D). These results suggest that FXR plays an anti-apoptotic role in HK2 cells and in our mouse model.

To test whether downregulation of FXR induces renal apoptosis, we transfected HK2 cells with siRNA to downregulate FXR and then exposed them to hypoxia. The Bax/Bcl-2 protein ratios in HK2 cells with FXR downregulation by siRNA were significantly increased in both normoxic and hypoxic HK2 cells compared with those of the siControl group (Fig. 5E). The hypoxia-induced increase of TUNEL-positive nuclei was markedly decreased by treatment with the FXR agonist GW4064 (Fig. 5F). Next, to determine whether dysregulation of renal autophagy induces renal apoptosis, we observed renal apoptosis in hypoxia-treated HK2 cells in which the autophagy-related protein ATG7 was downregulated by siRNA. The increase in the hypoxia-induced Bax/Bcl-2 protein ratio was further increased in normoxic and hypoxic HK2 cells in which ATG7 was downregulated, and the LC3 II/I ratio was also observed to be reduced compared with the control group (Supplementary Fig. 5A). Consistent with these results, the Bax/Bcl-2 protein ratios in the ATG7-downregulated cells exposed to normoxia and hypoxia-reoxygenation were significantly higher than those in the siControl group, and the LC3 II/I ratio was still decreased in the downregulated ATG7 group (Supplementary Fig. 5B). Taken together, these results suggest that dysregulation of autophagy induces renal apoptosis.

### FXR exerts an antioxidant effect

**Fig. 6:**
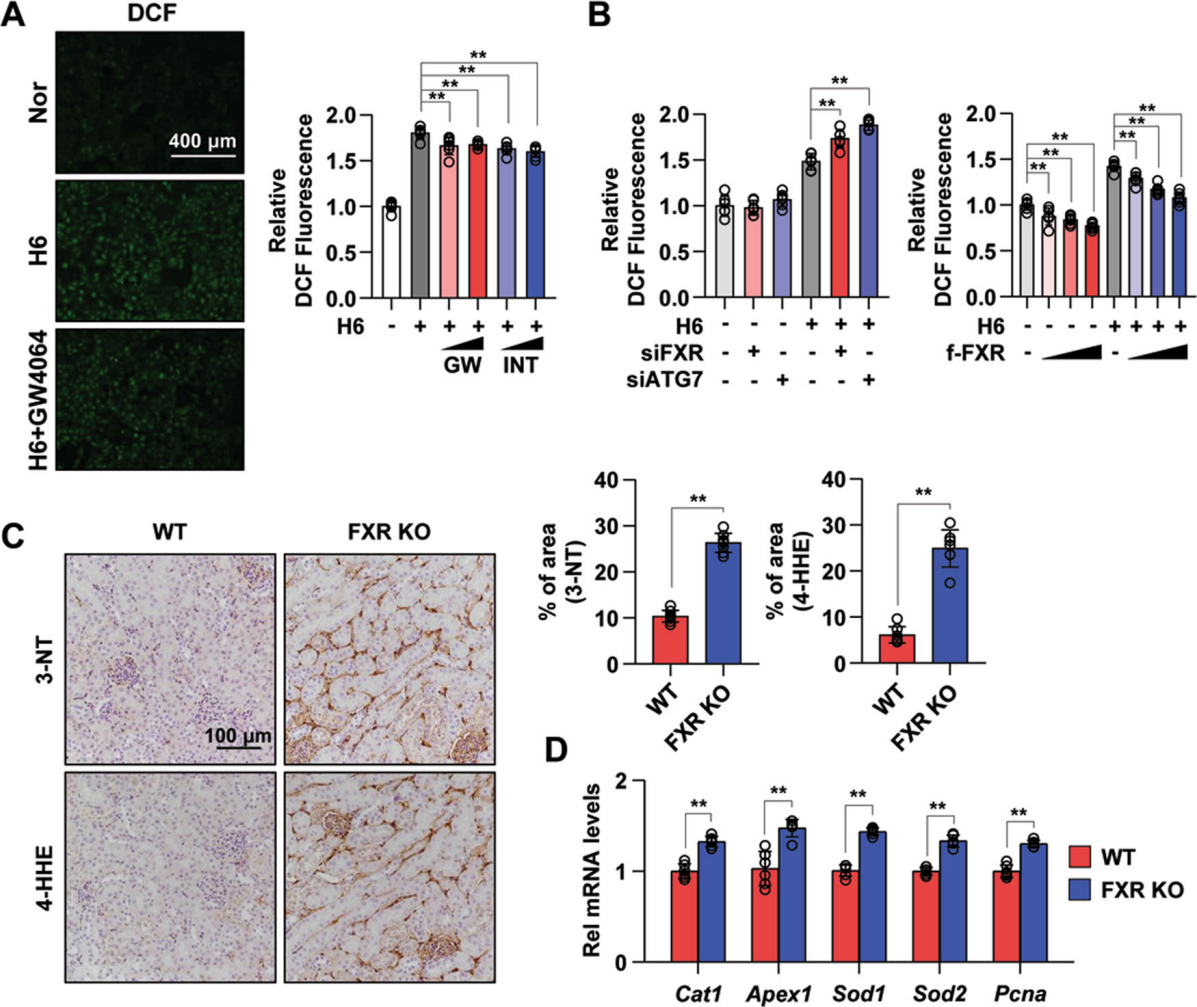
FXR activation inhibits ROS production. **A** After treatment with GW4064 (0.5 or 1 μM) or INT-747 (0.5 or 1 μM) for 1 h, HK2 cells were exposed to hypoxia for 6 h. The cells were labeled using CM-H_2_DCF-DA, and images were immediately visualized using the EVOS FL Auto Imaging System. Normoxic and hypoxia-exposed cells were incubated with CM-H_2_DCF-DA, and the ROS levels were measured using a Promega GloMax plate reader (right panel). The relative DCF fluorescence level is shown. The values for normoxia were set to 1 (*n* = 6). **B** HK2 cells were transfected with siFXR and siATG7 (left) or FXR expression plasmids (right) as indicated, and 48 h later, normoxic and hypoxia-exposed cells were incubated with CM-H_2_DCF-DA, and the ROS levels were measured. The values for normoxia of the siControl and vehicle were set to 1 (*n* = 6). **C** Paraffin-embedded kidney tissue sections from WT and FXR KO mice were stained with antibodies against 3-NT and 4-HHE (scale bar 100 μm). Computer-based morphometric analysis is shown (right bar graph, *n* = 7–8 in each group). **D** mRNA levels were detected by qRT-PCR (*n* = 6). All values are presented as the mean ± SD. Statistical significance was measured using one-way or two-way ANOVA with the Bonferroni post-test. *P < 0.05, **P < 0.005. 3-NT 3-nitrotyrosine, 4-HHE 4-hydroxy hexenal.

In a previous report that showed that FXR suppresses oxidative stress^30,31^, treatment with FXR agonist suppressed ROS production in mouse liver and kidney. Thus, we next measured ROS production in hypoxia-treated HK2 cells using a selective ROS probe, CM-H_2_DCF-DA. Increased ROS levels in hypoxia-treated HK2 cells were decreased by treatment with the FXR agonists GW4064 and INT-747 (Fig. 6A). Moreover, the downregulation of FXR and ATG7 by siRNA in hypoxia-treated HK2 cells further increased the ROS level compared with the vehicle-expressing group, and the ROS levels were significantly decreased in a dose-dependent manner by FXR overexpression in both normoxia-exposed and hypoxia-exposed HK2 cells (Fig. 6B). Immunostaining for 3-nitrotyrosine (3-NT) and 4-hydroxy hexenal (4-HHE) was performed in WT and FXR KO mouse kidneys to reveal ROS generation and lipid damage. The expression of 3-NT and 4-HHE was increased in FXR KO mouse kidneys compared with WT mouse kidneys, and the expression of genes involved in ROS production was significantly increased in FXR KO mouse kidneys compared with WT mouse kidneys (Fig. 6C, D). These data suggest that FXR is important for the regulation of ROS production in the kidney.

### FXR deficiency exacerbates the progression of AKI to CKD

**Fig. 7:**
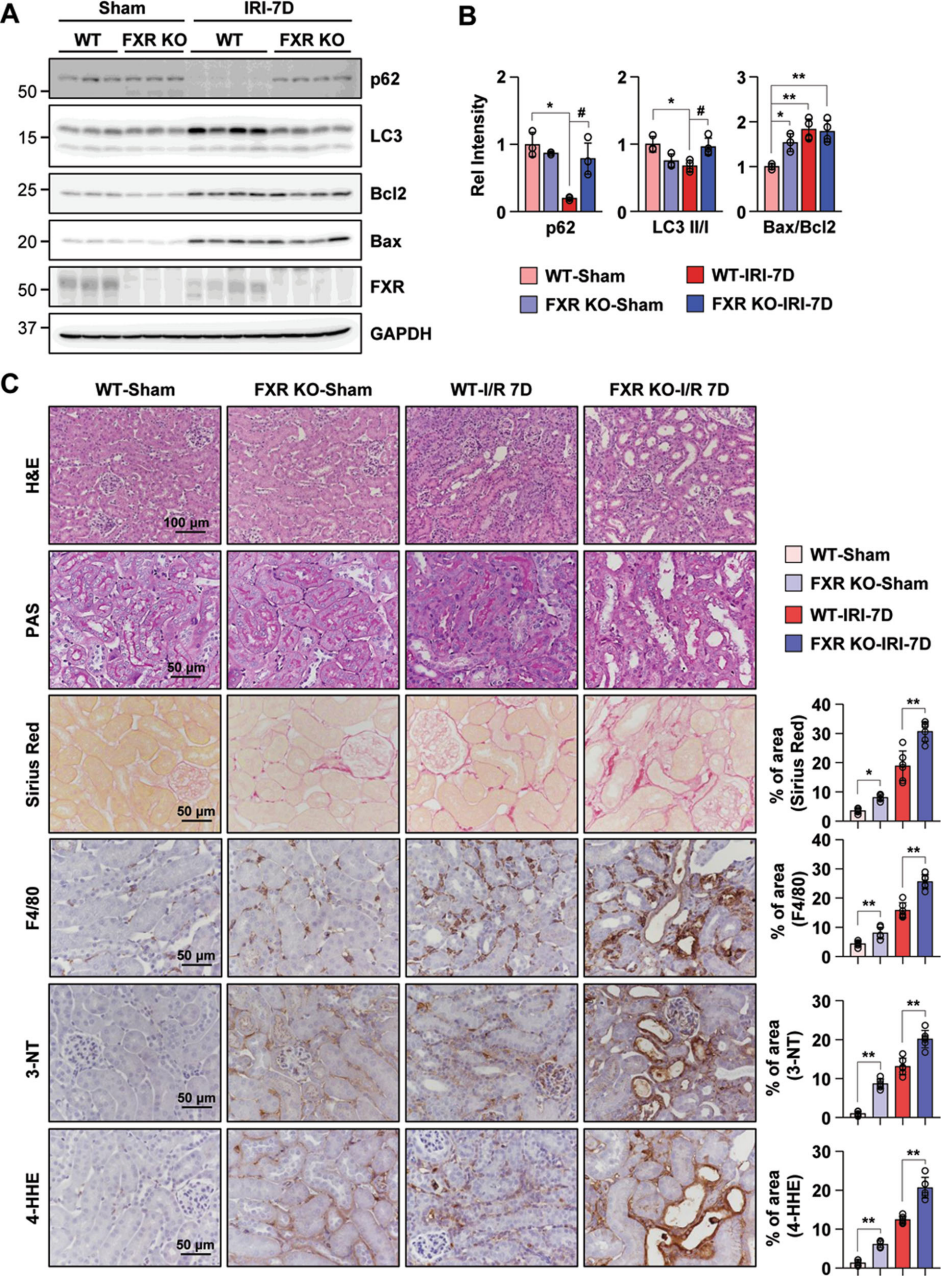
Loss of FXR increases renal damage in the sham and I/R injury mice after 7 days. At 7 days after I/R, kidney samples were collected for measurements. **A** Protein levels of p62, LC3, Bcl2, Bax, and FXR were detected by immunoblotting. **B** The relative protein levels are shown, and the values for the WT-Sham group were set to 1 (*n* = 3–4). **C** Renal cortical tissues were collected for hematoxylin and eosin (H&E), periodic acid-Schiff (PAS) and Sirius Red staining to examine histology and for immunohistochemistry to examine the expression of F4/80, 3-NT, and 4-HHE. Computer-based morphometric analysis is shown (right bar graph, *n* = 6 in each group). All values are presented as the mean ± SD. Statistical significance was measured using one-way or two-way ANOVA with the Bonferroni post-test. *P < 0.05, **P < 0.005, ^#^*P* < 0.05.

We examined whether the loss of FXR influences the progression of AKI in FXR KO mice. First, to test the renal dysfunction and damage of initial I/R injury, we monitored WT and FXR KO mice at 48 h after I/R injury. We examined the levels of serum blood urea nitrogen (BUN), creatinine (Cr), and neutrophil gelatinase-associated lipocalin (NGAL) for WT and FXR KO mice at 48 h after I/R injury. The levels of serum BUN, Cr, and NGAL in both WT and FXR KO in I/R injury mice were significantly increased compared with those of WT sham mice, and the levels of those in I/R injury groups did not differ between WT and FXR KO mice. In the tissue morphology, WT and FXR KO mice in I/R groups showed severe dilation of the proximal tubules, cast formation, and massive detachment of the tubular epithelium compared with WT sham group (Supplementary Fig. 6). Next, at 7 days after I/R injury, we tested the protein expression levels of p62, the LC3 II/I ratio, and the Bax/Bcl2 ratio in WT and FXR KO mice. The expression levels of p62 and the LC3 II/I ratio in WT mice in the I/R injury group were markedly decreased compared with those in the sham group, whereas the corresponding levels in FXR KO mice in the I/R injury group did not change. The Bax/Bcl2 ratio in WT mice in the I/R injury group and in FXR KO mice in the sham or I/R injury groups was markedly increased compared with that in WT mice in the sham group (Fig. 7A, B). In the tissue morphology, the FXR KO mice in the sham and I/R groups showed more severe tubular damage, which was characterized by detachment of tubular epithelial cells from the basement membranes and increased collagen deposition, compared with WT mice. Using immunohistochemistry, we examined F4/80, 3-NT, and 4-HHE expression to detect macrophage infiltration and ROS production. Compared with WT mice, FXR KO mice in both the sham and I/R injury groups exhibited a more marked increase in macrophage infiltration, ROS production, and lipid damage (Fig. 7C).

**Fig. 8:**
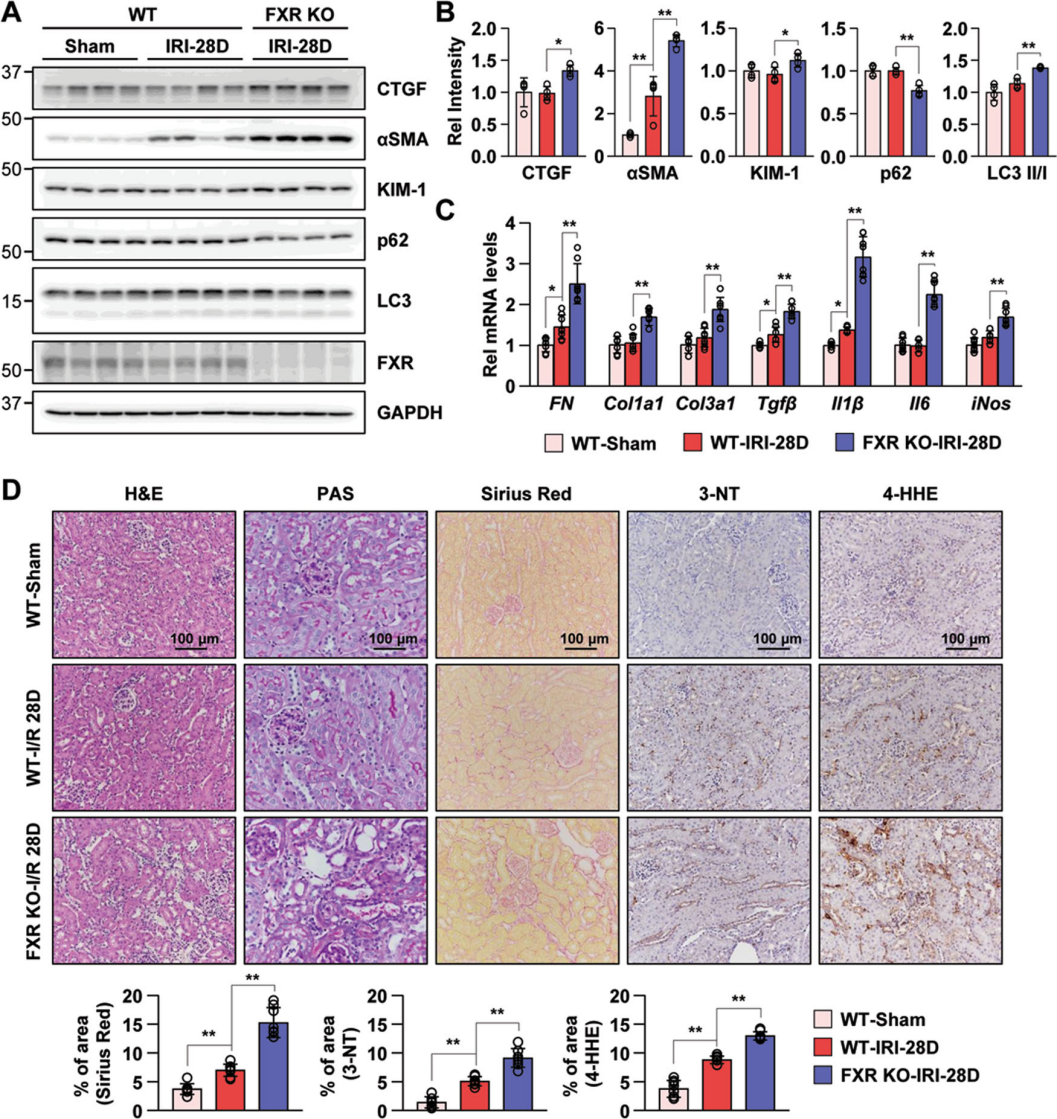
Loss of FXR exacerbates progression to CKD after I/R-induced AKI. At 28 days after I/R, kidney samples were collected for measurements. **A** Protein levels of CTGF, αSMA, KIM-1, p62, LC3, and FXR were detected by immunoblotting. **B** The relative protein levels are shown. The values for the WT-Sham group were set to 1 (*n* = 4). **C** mRNA levels were detected by qRT-PCR. The values for the WT-Sham were set to 1 (*n* = 8). **D** Renal cortical tissues were collected for hematoxylin and eosin (H&E), periodic acid-Schiff (PAS) and Sirius Red staining to examine histology and for immunohistochemistry to examine the expression of 3-NT and 4-HHE. Computer-based morphometric analysis is shown (bottom bar graph, *n* = 7–8 in each group). All values are presented as the mean ± SD. Statistical significance was measured using one-way or two-way ANOVA with the Bonferroni post-test. *P < 0.05, **P < 0.005.

To further examine whether the loss of FXR influences the progression of AKI to CKD, we monitored WT and FXR KO mice for 28 days after I/R injury. Compared with the WT mice in the I/R injury group, FXR KO mice in the I/R injury group showed significantly increased levels of NGAL (Supplementary Fig. 7A). We detected the protein expression levels of the fibrosis and kidney injury markers CTGF, αSMA, and KIM-1 to assess the progression of AKI to CKD. The levels of these indicators in mice in the FXR KO I/R injury group were markedly increased compared with those of WT mice in the I/R injury group, while the levels of CTGF and KIM-1 in mice in the WT I/R injury group did not change. The expression level of the p62 in FXR KO mice in the I/R injury group was significantly decreased compared with that in WT mice in the I/R injury group, while the LC3 II/I ratio and the levels of ATG3 and ATG7 were increased compared with those in WT mice. The Bax/Bcl2 ratio in both WT and FXR KO mice in the I/R injury group was decreased compared with that in WT mice in the sham group (Fig. 8A, B and Supplementary Fig. 7B). Consistent with these results, the mRNA levels of fibrosis-related genes (*Fibronectin*, *Col1a1*, and *Col3a1*) and inflammation-related genes (*Tgfβ*, *Il1β*, *Il6*, and *iNos*) in FXR KO mice in the I/R injury group were significantly higher than those in WT mice in the I/R injury group (Fig. 8C). At 28 days after I/R injury, WT mice showed increased renal tubulointerstitial damage, collagen deposits, peroxynitrite formation, and macrophage infiltration, as evidenced by histochemical stains and immunohistochemistry. Compared with WT mice, FXR KO mice in the I/R injury group exhibited a more marked increase in renal damage, fibrosis, ROS formation, and macrophage infiltration at 28 days (Fig. 8D and Supplementary Fig. 7C). Taken together, these data suggest that persistent activation of autophagy in FXR-deficient mice may trigger the renal cell death pathway and exaggerate renal damage, which would accelerate the progression to CKD.

## Discussion

In this study, we demonstrated that FXR is associated with the regulation of autophagy and apoptosis and that it was protective against the progression of AKI to CKD. We demonstrated that pharmacological activation of FXR regulates the increase in autophagy and apoptosis observed in hypoxia-treated tubular epithelial cells, and that FXR deficiency in both mice and cells increases the level of autophagy and apoptosis due to increased ROS levels. In addition, FXR deficiency significantly increases the expression levels of fibrosis markers, ROS, and autophagy, as observed over the long term after ischemic damage, which exacerbates the progression of AKI to CKD.

Several studies have reported that autophagy is induced by I/R injury in vivo and in vitro, and according to pharmacological and gene inhibitory studies, it plays a renoprotective role in AKI^14,32–34^. They suggested that basal autophagy is important for normal kidney homeostasis, and that renal dysfunction and damage were observed more in mouse kidneys that were deficient in autophagy-related gene expression. In addition, continuous autophagy activation exacerbates renal damage and triggers renal cell death pathways^35,36^. In the present study, we showed that increased autophagy and apoptosis levels in hypoxia-exposed proximal tubule cells are decreased by treatment with FXR agonists, and that FXR deficiency in cells and in mice increased the levels of autophagy, apoptosis, and oxidative stress. It has been reported that p62 exerts a protective effect in terms of ROS production and the DNA damage response in conditions of metabolic stress^37,38^. In our study, the p62 expression level in WT mice in the I/R injury group decreased on day 7 and recovered to a level similar to that in sham mice on day 28, whereas the p62 level in FXR KO mice did not. p62 in WT mice seems to play a role in reducing damage and ROS induced by the initial injury and in mitigating the progression to CKD, whereas this does not appear to be the case in FXR KO mice. Correlation studies on p62 and CKD progression are therefore needed in the future.

The activation of FXR is negatively correlated with the progression of obesity, diabetic nephropathy, and kidney I/R injury^19,39–41^. Feeding and fasting regulate the autophagy transcription by FXR and nutrient-related transcriptional factors, such as CREB and PPARα^12,27^. We showed that renal autophagy-related gene transcription levels are also regulated by nutrient sensing and FXR agonists, which is similar to what occurs in hepatic autophagy. We also showed that the levels of autophagy-related proteins and mRNAs in FXR-deficient mice were increased even in the absence of external stress. Previous studies have demonstrated the role of FXR-mediated protection from mitochondrial membrane peroxidation with antioxidant and free radical scavenging activity, which occurs through FXR activation^31,42–44^. Our data showed that the level of ROS was increased in FXR-deficient mice and cells and that 7 days after I/R injury, the ROS level in FXR KO mice was further increased compared with that in WT mice. After 28 days of I/R injury, the ROS level in WT mice in our model recovered to a level similar to that in sham mice but was still increased in FXR KO mice, which seemed to exacerbate renal fibrosis and inflammation. The results of the present study suggest that the potential importance of FXR for the negative regulation of renal autophagy and ROS.

Autophagy and apoptosis occur as a result of various stress pathways; generally, autophagy blocks the induction of apoptosis, and the activation of apoptosis-related caspases interrupts the autophagic process^45^. The role of pharmacological activation of FXR was reported to ameliorate liver injury and induce apoptosis in cells and in vivo^46–48^. In addition, FXR agonists were shown to suppress renal apoptosis and damage in conditions associated with ischemic kidney injury^31^. In the present study, apoptosis in the kidney was increased in ischemic injury, and FXR deficiency in mouse kidneys increased renal apoptosis. In long-term I/R injury, the level of apoptosis did not differ between the WT and FXR KO mice in our model; however, the autophagy level in the FXR KO mice continued to increase. For this reason, the FXR KO mice exhibited increased renal fibrosis, inflammation, and progression to CKD compared with the WT mice in our model, and further research on the cell death pathways and pharmacological inhibitors of autophagy and apoptosis in this FXR KO mouse model is needed in the future.

This study suggests that the activation of FXR by pharmacological means or by nutrient sensing protects against renal damage and fibrosis through the regulation of renal autophagy, apoptosis, and ROS production. In the early stages of ischemic injury, FXR regulates autophagy, which induces apoptosis to eliminate dying cells and restore normal conditions. Moreover, this finding suggests a mechanistic basis for the regulation of autophagy and apoptosis by activation of FXR, which may be a potential pharmacological target in the early stages of kidney damage and as a therapy when AKI progresses to CKD.

## Supplementary information

Supplementary Figure Legends

Supplementary Table 1

Supplementary Figure 1

Supplementary Figure 2

Supplementary Figure 3

Supplementary Figure 4

Supplementary Figure 5

Supplementary Figure 6

Supplementary Figure 7

## References

[1] Chertow GM, Burdick E, Honour M, Bonventre JV, Bates DW (2005). Acute kidney injury, mortality, length of stay, and costs in hospitalized patients. J. Am. Soc. Nephrol..

[2] Ronco C, Bellomo R, Kellum JA (2019). Acute kidney injury. Lancet.

[3] Kaushal GP, Shah SV (2016). Autophagy in acute kidney injury. Kidney Int..

[4] Tang C, Livingston MJ, Liu Z, Dong Z (2020). Autophagy in kidney homeostasis and disease. Nat. Rev. Nephrol..

[5] Humphreys BD (2008). Intrinsic epithelial cells repair the kidney after injury. Cell Stem Cell.

[6] Levine B, Klionsky DJ (2004). Development by self-digestion: molecular mechanisms and biological functions of autophagy. Dev. Cell.

[7] Mizushima N, Komatsu M (2011). Autophagy: renovation of cells and tissues. Cell.

[8] Levine B, Kroemer G (2008). Autophagy in the pathogenesis of disease. Cell.

[9] Majmundar AJ, Wong WJ, Simon MC (2010). Hypoxia-inducible factors and the response to hypoxic stress. Mol. Cell.

[10] Meijer AJ, Codogno P (2011). Autophagy: regulation by energy sensing. Curr. Biol..

[11] Settembre C (2013). TFEB controls cellular lipid metabolism through a starvation-induced autoregulatory loop. Nat. Cell Biol..

[12] Seok S (2014). Transcriptional regulation of autophagy by an FXR-CREB axis. Nature.

[13] Scherz-Shouval, R. et al. Reactive oxygen species are essential for autophagy and specifically regulate the activity of Atg4. *EMBO J.* **26**, 38 (2019). 10.15252/embj.2019101812 PMC6517991 31092559

[14] Kimura T (2011). Autophagy protects the proximal tubule from degeneration and acute ischemic injury. J. Am. Soc. Nephrol..

[15] Jiang M (2012). Autophagy in proximal tubules protects against acute kidney injury. Kidney Int..

[16] Liu S (2012). Autophagy plays a critical role in kidney tubule maintenance, aging and ischemia-reperfusion injury. Autophagy.

[17] Modica S, Gadaleta RM, Moschetta A (2010). Deciphering the nuclear bile acid receptor FXR paradigm. Nucl. Recept. Signal..

[18] Calkin AC, Tontonoz P (2012). Transcriptional integration of metabolism by the nuclear sterol-activated receptors LXR and FXR. Nat. Rev. Mol. Cell Biol..

[19] Jiang T (2007). Farnesoid X receptor modulates renal lipid metabolism, fibrosis, and diabetic nephropathy. Diabetes.

[20] Hu Z, Ren L, Wang C, Liu B, Song G (2012). Effect of chenodeoxycholic acid on fibrosis, inflammation and oxidative stress in kidney in high-fructose-fed Wistar rats. Kidney Blood Press. Res..

[21] Zhao K (2016). Activation of FXR protects against renal fibrosis via suppressing Smad3 expression. Sci. Rep..

[22] Kim DH (2019). Src-mediated crosstalk between FXR and YAP protects against renal fibrosis. FASEB J..

[23] Kimura S, Noda T, Yoshimori T (2007). Dissection of the autophagosome maturation process by a novel reporter protein, tandem fluorescent-tagged LC3. Autophagy.

[24] Kim DH (2015). A dysregulated acetyl/SUMO switch of FXR promotes hepatic inflammation in obesity. EMBO J..

[25] Kim CS (2016). Nicotine-induced apoptosis in human renal proximal tubular epithelial cells. PLoS ONE.

[26] Kim J, Kundu M, Viollet B, Guan KL (2011). AMPK and mTOR regulate autophagy through direct phosphorylation of Ulk1. Nat. Cell Biol..

[27] Lee JM (2014). Nutrient-sensing nuclear receptors coordinate autophagy. Nature.

[28] Kaushal GP, Singh AB, Shah SV (1998). Identification of gene family of caspases in rat kidney and altered expression in ischemia-reperfusion injury. Am. J. Physiol..

[29] Basnakian AG, Ueda N, Kaushal GP, Mikhailova MV, Shah SV (2002). DNase I-like endonuclease in rat kidney cortex that is activated during ischemia/reperfusion injury. J. Am. Soc. Nephrol..

[30] Wang YD (2015). Farnesoid X receptor antagonizes JNK signaling pathway in liver carcinogenesis by activating SOD3. Mol. Endocrinol..

[31] Gai Z (2017). Farnesoid X receptor activation protects the kidney from ischemia-reperfusion damage. Sci. Rep..

[32] Jiang M, Liu K, Luo J, Dong Z (2010). Autophagy is a renoprotective mechanism during in vitro hypoxia and in vivo ischemia-reperfusion injury. Am. J. Pathol..

[33] Li J (2016). Metformin protects against cisplatin-induced tubular cell apoptosis and acute kidney injury via AMPKalpha-regulated autophagy induction. Sci. Rep..

[34] Poluzzi C (2019). Biglycan evokes autophagy in macrophages via a novel CD44/Toll-like receptor 4 signaling axis in ischemia/reperfusion injury. Kidney Int..

[35] Decuypere JP (2015). Autophagy and the kidney: implications for ischemia-reperfusion injury and therapy. Am. J. Kidney Dis..

[36] Duann P., Lianos, E. A., Ma, J. & Lin, P. H. Autophagy, innate immunity and tissue repair in acute kidney injury. *Int. J. Mol. Sci.* **17**, 662 (2016). 10.3390/ijms17050662 PMC4881488 27153058

[37] Mathew R (2009). Autophagy suppresses tumorigenesis through elimination of p62. Cell.

[38] Liao W (2019). p62/SQSTM1 protects against cisplatin-induced oxidative stress in kidneys by mediating the cross talk between autophagy and the Keap1-Nrf2 signalling pathway. Free Radic. Res..

[39] Huls M, van den Heuvel JJ, Dijkman HB, Russel FG, Masereeuw R (2006). ABC transporter expression profiling after ischemic reperfusion injury in mouse kidney. Kidney Int..

[40] Proctor G (2006). Regulation of renal fatty acid and cholesterol metabolism, inflammation, and fibrosis in Akita and OVE26 mice with type 1 diabetes. Diabetes.

[41] Wang XX (2010). Diabetic nephropathy is accelerated by farnesoid X receptor deficiency and inhibited by farnesoid X receptor activation in a type 1 diabetes model. Diabetes.

[42] Livero FA (2014). The FXR agonist 6ECDCA reduces hepatic steatosis and oxidative stress induced by ethanol and low-protein diet in mice. Chem. Biol. Interact..

[43] Wu WB (2015). Agonist of farnesoid X receptor protects against bile acid induced damage and oxidative stress in mouse placenta—a study on maternal cholestasis model. Placenta.

[44] Gai Z, Gui T, Hiller C, Kullak-Ublick GA, Farnesoid X (2016). Receptor protects against kidney injury in uninephrectomized obese mice. J. Biol. Chem..

[45] Marino G, Niso-Santano M, Baehrecke EH, Kroemer G (2014). Self-consumption: the interplay of autophagy and apoptosis. Nat. Rev. Mol. Cell Biol..

[46] Wang YD (2008). Farnesoid X receptor protects liver cells from apoptosis induced by serum deprivation in vitro and fasting in vivo. Mol. Endocrinol..

[47] Lian F (2015). Activated farnesoid X receptor attenuates apoptosis and liver injury in autoimmune hepatitis. Mol. Med. Rep..

[48] Wang H (2018). Noncanonical farnesoid X receptor signaling inhibits apoptosis and impedes liver fibrosis. EBioMedicine.

