# Supplementary material for: The critical role of FXR is associated with the regulation of autophagy and apoptosis in the progression of AKI to CKD
Source: Cell Death Dis. 2021 Mar 25;12(4):320. doi: 10.1038/s41419-021-03620-z (PMC7994637; doi:10.1038/s41419-021-03620-z)
Supplement: Supplementary file 1 — Supplementary Figure Legends [file 41419_2021_3620_MOESM1_ESM.docx]

**Supplementary Figure 1. The effects of FXR agonists and antagonist in hypoxia-induced HK2 cells.**

After treatment with z-guggulsterone (15 μM), GW4064 (1 μM), or INT-747 (1 μM) for 1 h, HK2 cells were exposed to hypoxia for another 6 h. The mRNA levels of autophagy-related genes*, ATG2A, ATG2B, ATG7*, and *ULK1*, were measured by qRT-PCR. The values for vehicle normoxia treatment were set to 1 (n = 4). All values are presented as mean ± SD. Statistical significance was measured using the one-way ANOVA with the Bonferroni post-test. **P<0.005 and ^##^P<0.005.

**Supplementary Figure 2. Deficient of FXR increased autophagy flux in hypoxia-induced HK2 cells.**

HK2 cells were transfected with the siFXR as indicated, and 48 h later, the cells were exposed to hypoxia for 6 h after autophagy inhibitors (Baf-1 and CQ) treatment for 1 h. The protein levels of the indicated genes were detected by immunoblotting (A), and the relative protein levels are shown (n = 4). The values for the siControl normoxia were set to 1. All values are presented as the mean ± SD. Statistical significance was measured using one-way ANOVA with the Bonferroni post-test. *P<0.05, **P<0.005.

**Supplementary Figure 3. Renal apoptosis is induced in I/R injury and hypoxia-induced HK2 cells.**

(A) At 48 h after I/R, the mice were euthanized and the kidneys were collected (n = 4). Protein levels of Bcl2 and Bax were detected by immunoblot. Relative protein levels were shown. The values for Sham group are set to 1. (B) HK2 cells were incubated with hypoxia for indicated time and protein levels of Bcl2 and Bax were detected by immunoblot. Relative levels are shown (n=3). Each Bax/Bcl2 levels of hypoxia 0 h sample were set as 1. All values are presented as mean ± SD. Statistical significance was measured using the one-way ANOVA with the Bonferroni post-test. *P<0.05, **P<0.005.

**Supplementary Figure 4. Pharmacological activation of FXR increased the cell viability in hypoxia-induced HK2 cells.**

(A and B) After treatment with GW4064 (0.25 to 1 μM) or WAY-362450 (0.5 and 1 μM) for 1 h, HK2 cells were incubated with hypoxia for 6 h (A) or reoxygenation for 20 h (B) (n = 4 – 6). Cell viability was determined using the EZ-CyTox. All values are presented as mean ± SD. Statistical significance was measured using the one-way ANOVA with the Bonferroni post-test. *P<0.05, **P<0.005.

**Supplementary Figure 5. ATG7 deficiency increases renal apoptosis.**

HK2 cells were transfected with siRNA for ATG7 as indicated, and 48 h later, the cells were exposed to hypoxia for 6 h or reoxygenation for 20 h. The protein levels of the indicated gene were detected by immunoblot. Relative intensity was shown. The value for siControl normoxic is set to 1 (n=3). All values are presented as mean ± SD. Statistical significance was measured using the two-way ANOVA with the Bonferroni post-test. *P<0.05, **P<0.005, ^##^P<0.005.

**Supplementary Figure 6. Renal dysfunction and tissue damage due to initial I/R injury were increased in both WT and FXR KO mice.**

At 48 hours after I/R, kidney samples were collected for measurements. (A) Blood urea nitrogen (BUN), creatinine (Cr), and neutrophil gelatinase-associated lipocalin (NGAL) levels were measured in serum (n = 5 – 7). (B) Paraffin–embedded kidney tissue sections from sham or I/R mice were performed hematoxylin and eosin (H&E) and periodic acid-Schiff (PAS) (scale bar 100 μm).

**Supplementary Figure 7. Loss of FXR increases renal fibrosis in sham and I/R mice model.**

At 28 days after I/R, kidney samples were collected for measurements. (A) Neutrophil gelatinase-associated lipocalin (NGAL) levels were measured in serum (n = 5 – 7). (B) Protein levels of ATG3, ATG7, Bcl2, and Bax were detected by immunoblot. Relative protein levels were shown. The values for WT-Sham group are set to 1 (n = 4). Paraffin–embedded kidney tissue sections from sham or I/R mice were performed F4/80 and Collagen IV. (scale bar 100 μm). Computer-based morphometric analysis is shown (Right bar graph, n = 8 in each group).
