# Supplementary material for: The critical role of FXR is associated with the regulation of autophagy and apoptosis in the progression of AKI to CKD
Source: Cell Death Dis. 2021 Mar 25;12(4):320. doi: 10.1038/s41419-021-03620-z (PMC7994637; doi:10.1038/s41419-021-03620-z)
Supplement: Supplementary file 2 — Supplementary Table 1 [file 41419_2021_3620_MOESM2_ESM.docx]

**Supplementary Table 1. List of primer sequences for qRT-PCR.**

|  | **Forward** | **Reverse** |
| --- | --- | --- |
| mAtg2a | CACTCTACGCCACTACAT | ATCCAGCACATCCAAGAA |
| mAtg2b | CCTCCACTCTCAGAATCA | GTCCATCACACGAACATAA |
| mAtg3 | TCACAACACAGGTATTACAG | CTTCCTCGTCTTCTTCATC |
| mAtg5 | ACTTGCTTTACTCTCTATCAG | CATCTTCTTGTCTCATAACCT |
| mAtg7 | CAGAAGAAGTTGAACGAGTA | CAGAGTCACCATTGTAGTAAT |
| mUlk1 | ACCATTGTCTACCAGTGT | AGTGTCTTGTTCTTCTCATAA |
| mBecn1 | ATGTGGAAAAGAACCGCAAG | ACTCCAGCTGCTGCCTTTTA |
| mLc3a | GCCTGTCCTGGATAAGACCA | GGTTGACCAGCAGGAAGAAG |
| mLc3b | TTCTTCCTCCTGGTGAATGG | TGCGAGGCATAAACCATGTA |
| mp62 | TCGGAAGCTGAAACATGGAC | GACTCAGCTGTAGGGCAAGG |
| mShp | TCTGCAGGTCGTCCGACTAT | CAGGCAGTGGCTGTGAGAT |
| mBsep | CAATGTTCAGTTCCTCCGTTCA | TTTGGTGTTGTCCCCATACTTG |
| mOstα | GTTGCCATTTTTCTGGAGGA | GACCAAAGCAGCAGAACACA |
| mOstβ | ATCCTGGCAAACAGAAATCG | GGCCAAGTCTGGTTTCTCTG |
| mCat1 | CCTCGTTCAGGATGTGGTTT | TCTGGTGATATCGTGGGTGA |
| mApex1 | GCTCCGTCAGACAAAGAAGG | GCATTGGGAACATAGGCTGT |
| mSod1 | CCAGTGCAGGACCTCATTTTA | TCACACGATCTTCAATGGACA |
| mSod2 | GGCCTACGTGAACAATCTCAA | TCAGGTTTGTCCAGAAAATGG |
| mPcna | GACTCGTCTCACGTCTCCTTG | AGAATTTTGGACATGCTGGTG |
| mCol1a1 | GAGCGGAGAGTACTGGATCG | AGACGGCTGAGTAGGGAACA |
| mCol3a1 | ACCAAAAGGTGATGCTGGAC | GACCTCGTGCTCCAGTTAGC |
| mIl1β | AACCTGCTGGTGTGTGACGTTC | CAGCACGAGGCTTTTTTGTTGT |
| mIl6 | AGAAGGAGTGGCTAAGGACCAA | AACGCACTAGGTTTGCCGAGTA |
| m36B4 | CCCTGAAGTGCTCGACATCA | TGCGGACACCCTCCAGAA |
| hAtg2a | CGACGTGCCCACTCTAACAT | GGCATCCTGGTCCACATTGA |
| hAtg2b | TCACACACCCACGAGACATG | TTTCATGGCGGGTAGACTT |
| hAtg3 | CAATGGGCTACAGGGGAAGA | ATTCCATCTGTTTGCACCGC |
| hAtg7 | ATGATCCCTGTAACTTAGCCCA | CACGGAAGCAAACAACTTCAAC |
| hLc3b | GCCTTCTTCCTGTTGGTGAA | CTGGGAGGCATAGACCATGT |
| hFxr | AAACAGAGGATGCCTCAGGA | GTTGCCATTTCCGTCAAAAT |
| h36B4 | TGCTGAACATGCTCAAC | GTCGAACACCTGCTGGATGAC |
