# Supplementary figures and images for: The critical role of FXR is associated with the regulation of autophagy and apoptosis in the progression of AKI to CKD
Source: Cell Death Dis. 2021 Mar 25;12(4):320. doi: 10.1038/s41419-021-03620-z (PMC7994637; doi:10.1038/s41419-021-03620-z)

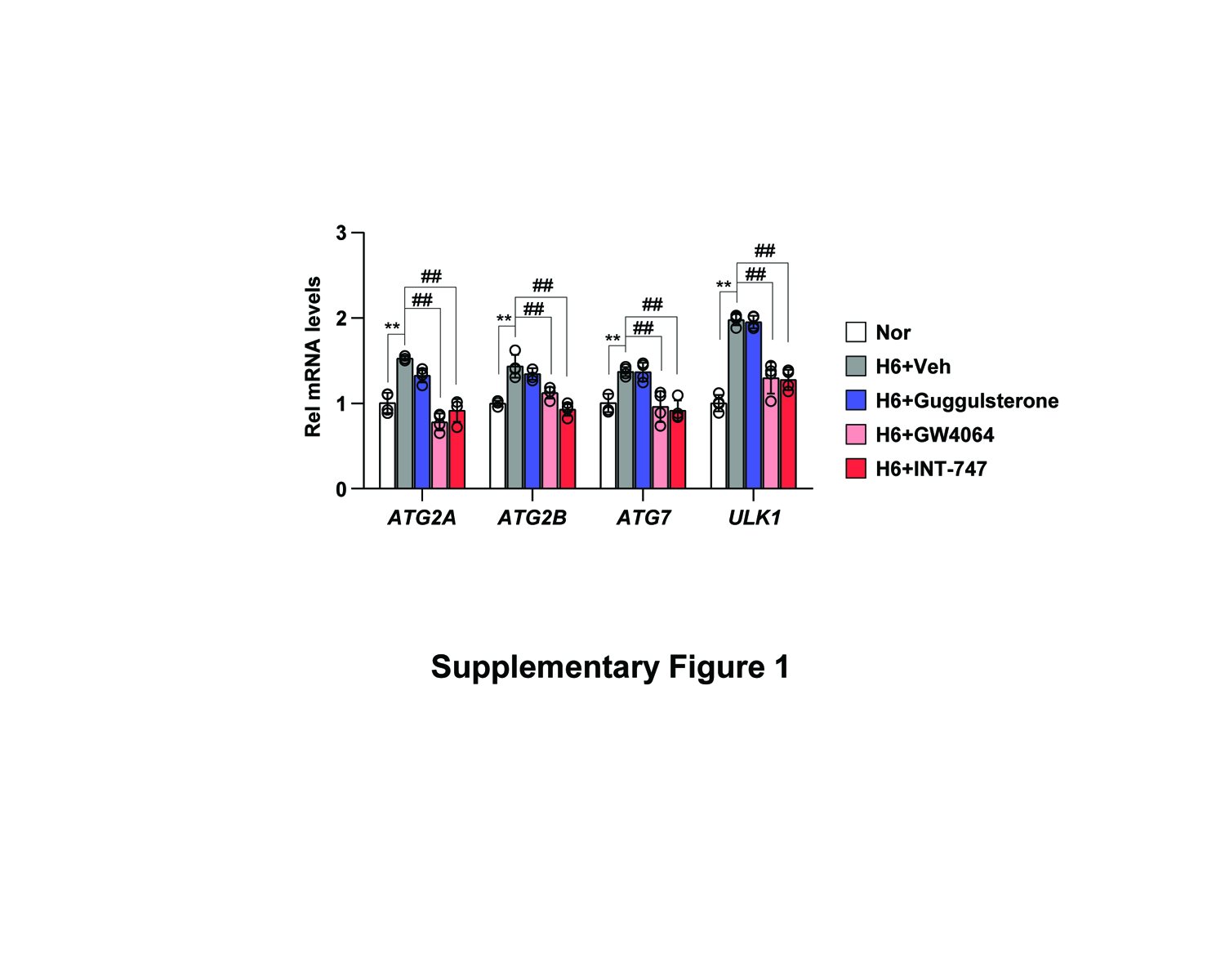

Supplement: Supplementary file 3 — Supplementary Figure 1 [file 41419_2021_3620_MOESM3_ESM.tif]

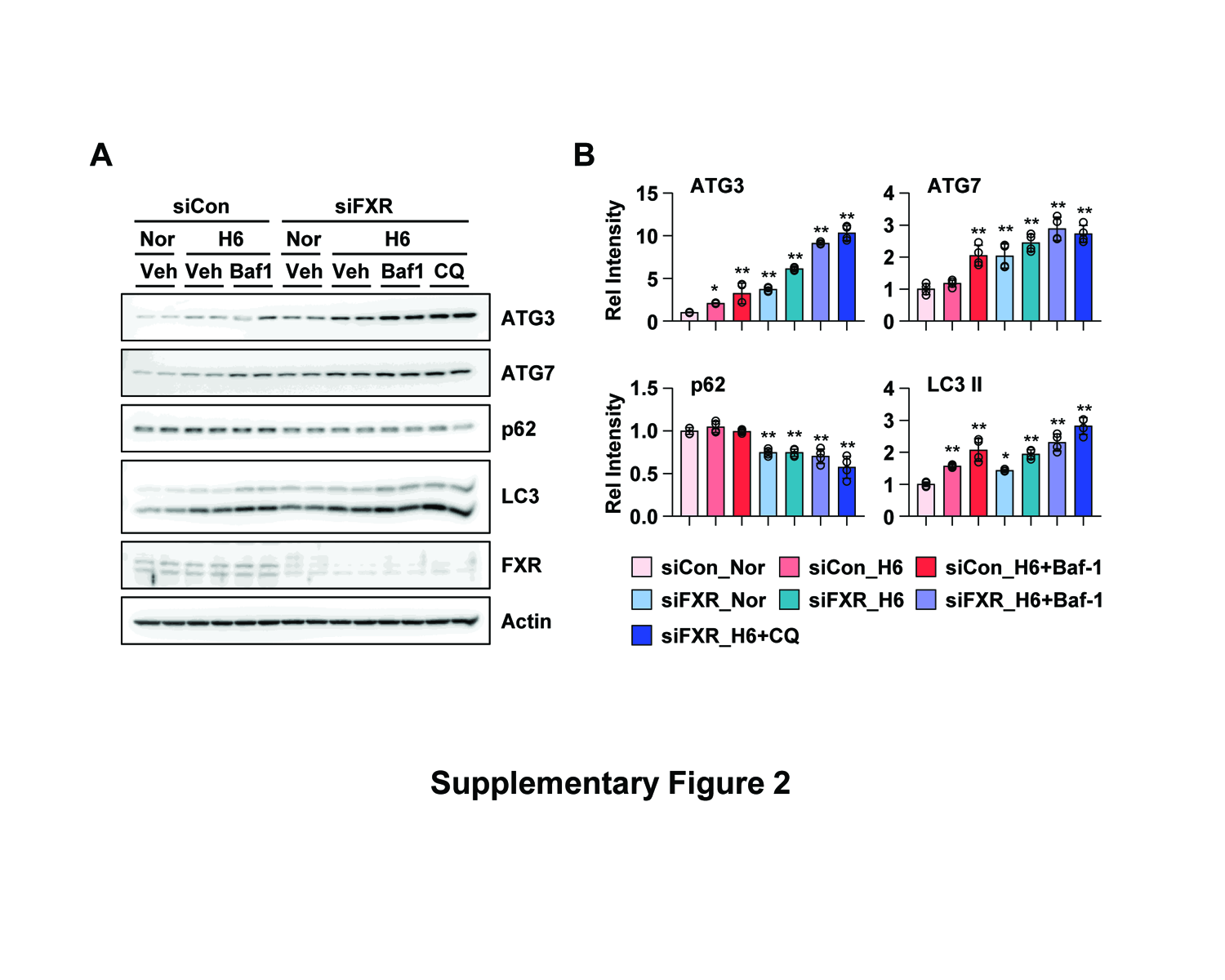

Supplement: Supplementary file 4 — Supplementary Figure 2 [file 41419_2021_3620_MOESM4_ESM.tif]

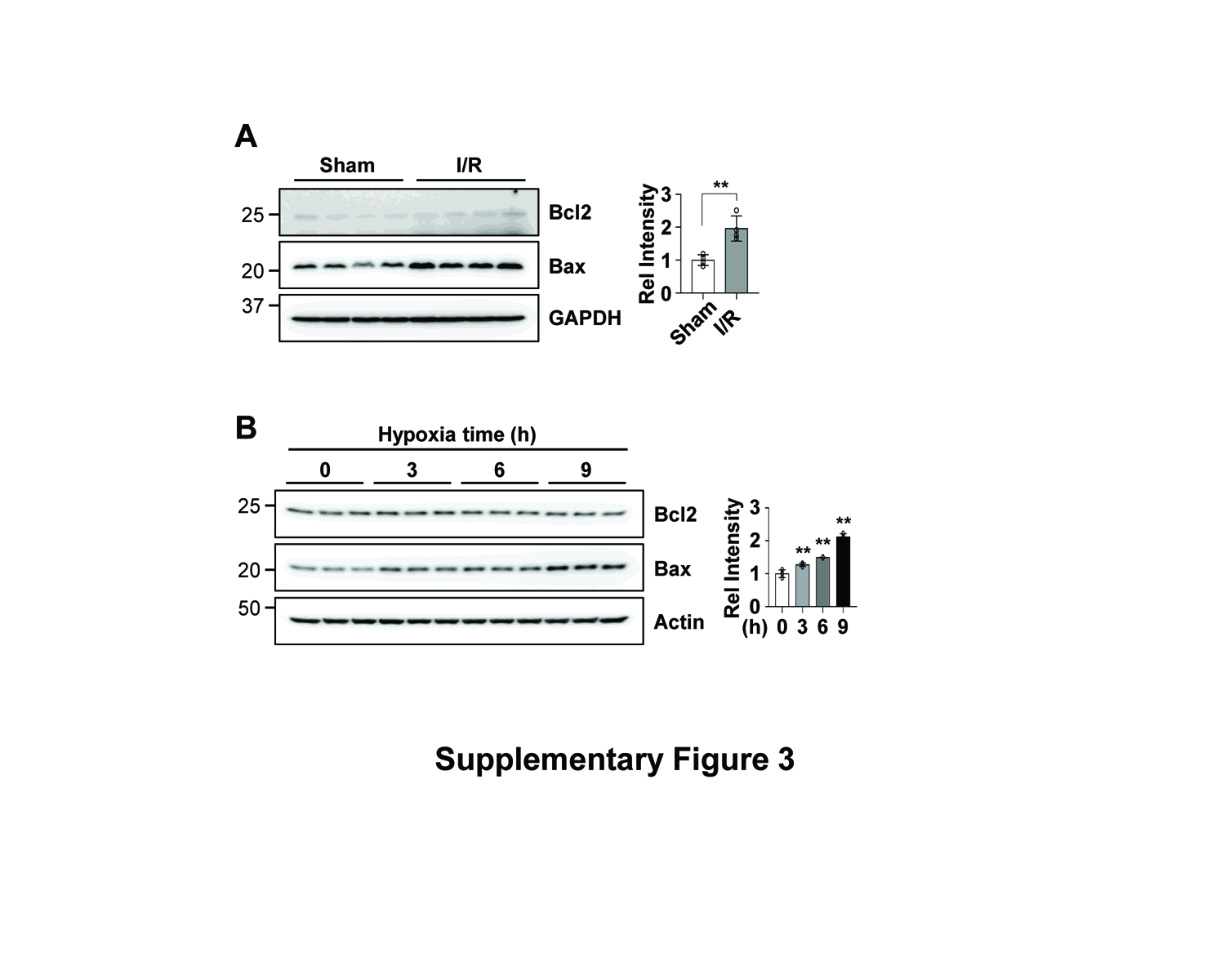

Supplement: Supplementary file 5 — Supplementary Figure 3 [file 41419_2021_3620_MOESM5_ESM.tif]

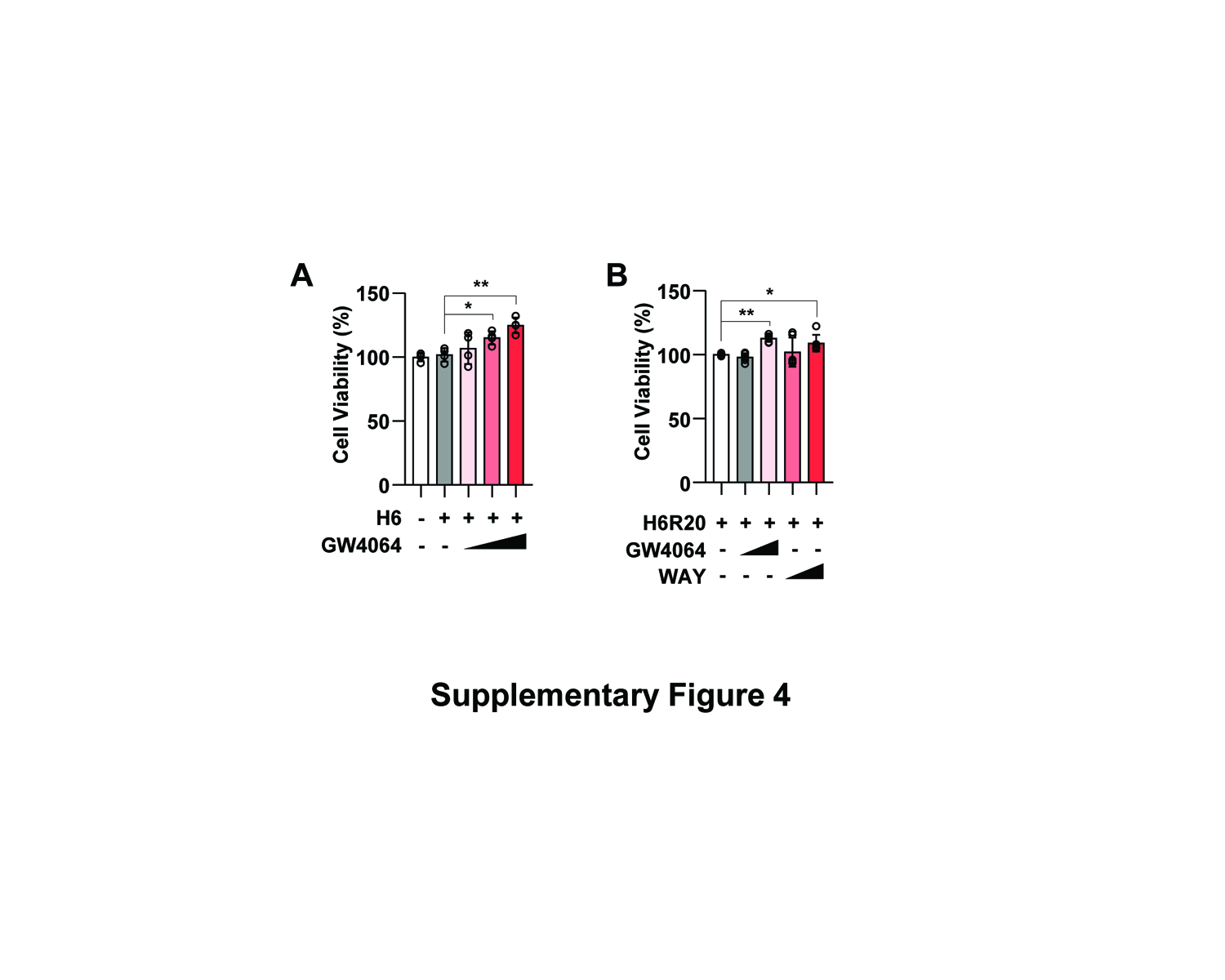

Supplement: Supplementary file 6 — Supplementary Figure 4 [file 41419_2021_3620_MOESM6_ESM.tif]

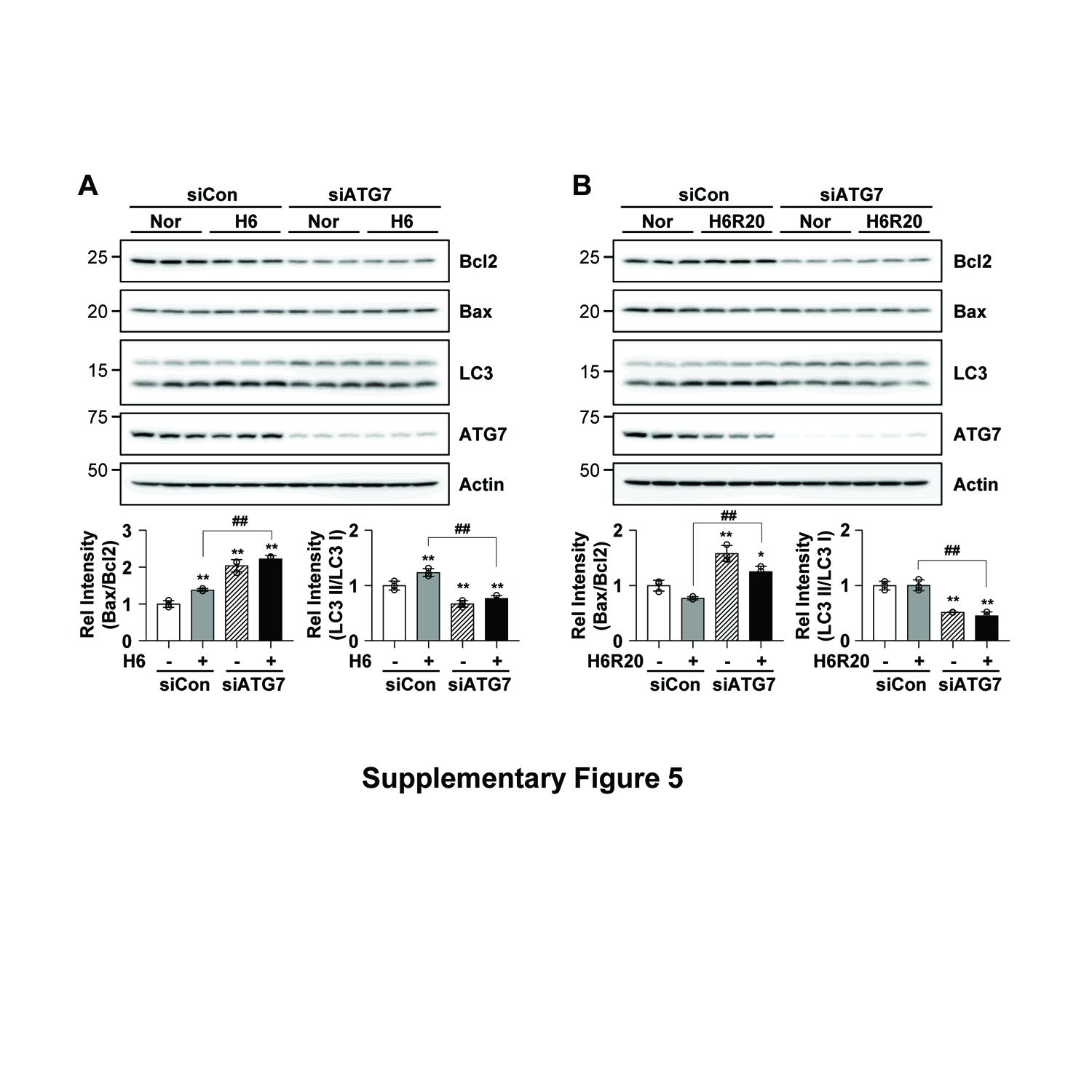

Supplement: Supplementary file 7 — Supplementary Figure 5 [file 41419_2021_3620_MOESM7_ESM.tif]

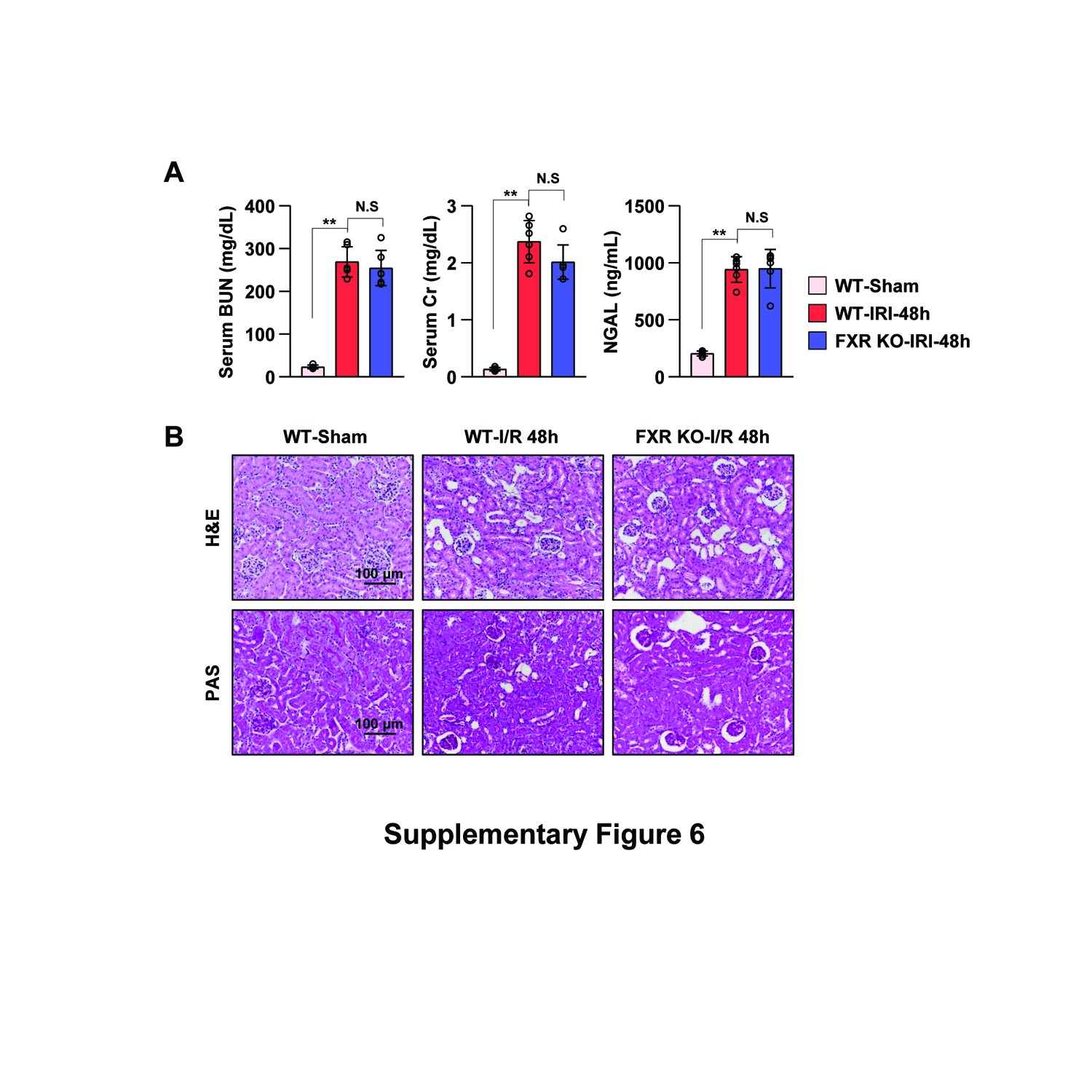

Supplement: Supplementary file 8 — Supplementary Figure 6 [file 41419_2021_3620_MOESM8_ESM.tif]

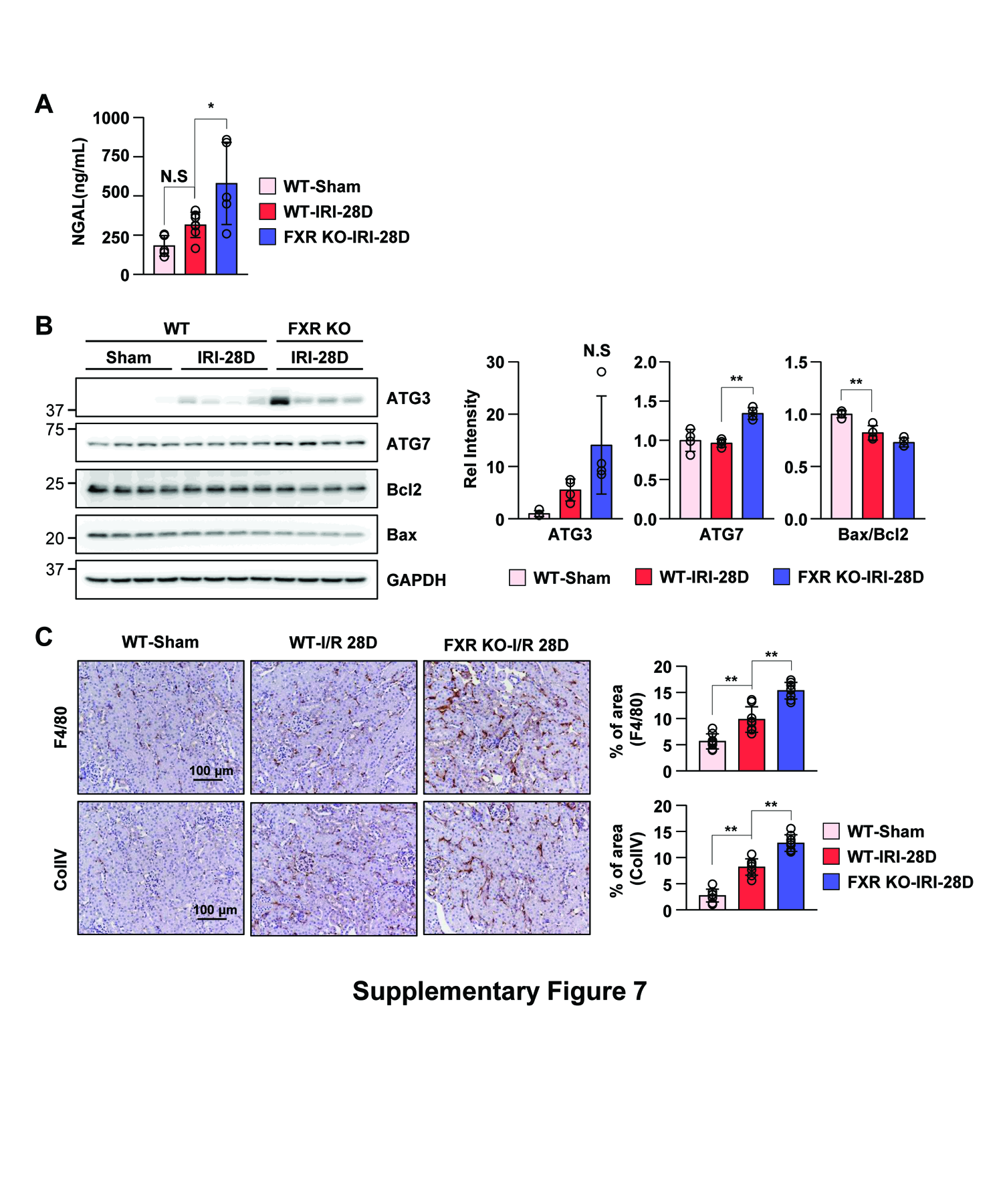

Supplement: Supplementary file 9 — Supplementary Figure 7 [file 41419_2021_3620_MOESM9_ESM.tif]
